# Formation of 3-Oxa- and 3-Thiacyclohexyne from Ring Expansion of Heterocyclic Alkylidene Carbenes: A Mechanistic Study

**DOI:** 10.1021/acs.orglett.3c00042

**Published:** 2023-03-01

**Authors:** T. E. Anderson, Dasan M. Thamattoor, David Lee Phillips

**Affiliations:** † Department of Chemistry, Colby College, 5765 Mayflower Hill, Waterville, Maine 04901, USA; ‡ Department of Chemistry, The University of Hong Kong, Pokfulam Road, Pok Fu Lam 999077, Hong Kong S.A.R.

## Abstract

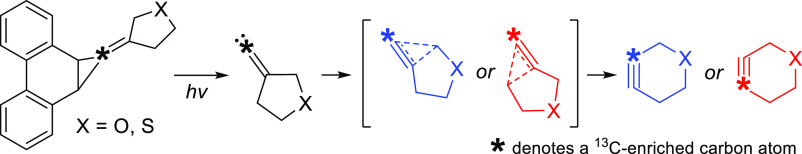

The rearrangement pathways of two alkylidene carbenes appended to an oxa or thiacyclopentane into the corresponding heterocyclohexynes were elucidated using ^13^C-labeling experiments. Both carbenes exhibited a preference for migration of the allylic carbon bound to the heteroatom. Anomeric interactions involving a heteroatom lone pair and antibonding orbital of the migrating bond and inductive destabilization of the minor migratory pathway are discussed as plausible reasons for the observed trends.

Small-ring strained cycloalkynes, which exhibit unusually large distortion around the sp-hybridized carbon atoms within the ring, are valuable molecules for both practical and theoretical reasons.^1−5^ The incorporation of heteroatoms into the ring structures of cycloalkynes enhances the utility of these molecules by allowing for the modulation of click reactivity^6−9^ and the expansion of methodologies for natural product synthesis.^10,11^ The generation of heterocycloalkynes composed of seven or fewer atoms is difficult, however, due to their instability under ambient conditions.^12^ Piperidynes **1**(13) and **2**,^14^ along with oxacyclohexyne **3**, which has been synthesized by Garg and co-workers^15^ and more recently in our own laboratory,^16^ are among the only small-ring heterocycloalkynes that have been prepared to date (Figure 1). To our knowledge, the generation of thiacyclohexyne **4** has not been reported prior to this work.

**Figure 1:**
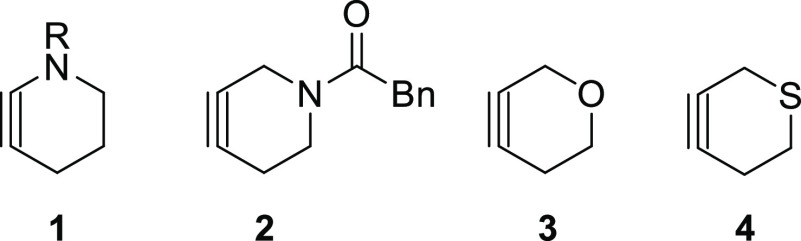
Previously generated small-ring heterocycloalkynes.

Our synthesis of oxacyclohexyne **3** was accomplished through the irradiation of phenanthrene derivative **5** with ultraviolet light (Scheme 1).^16^ Photolysis of **5** generates the exocyclic alkylidene carbene **7**, which undergoes a Fritsch–Buttenberg–Wiechell (FBW)-type rearrangement to form **3**.^16−21^ The reactive alkyne was intercepted through a Diels–Alder cycloaddition reaction with cyclopentadienone **8**, yielding adduct **9** after the loss of carbon monoxide.^16^

**Scheme 1:**
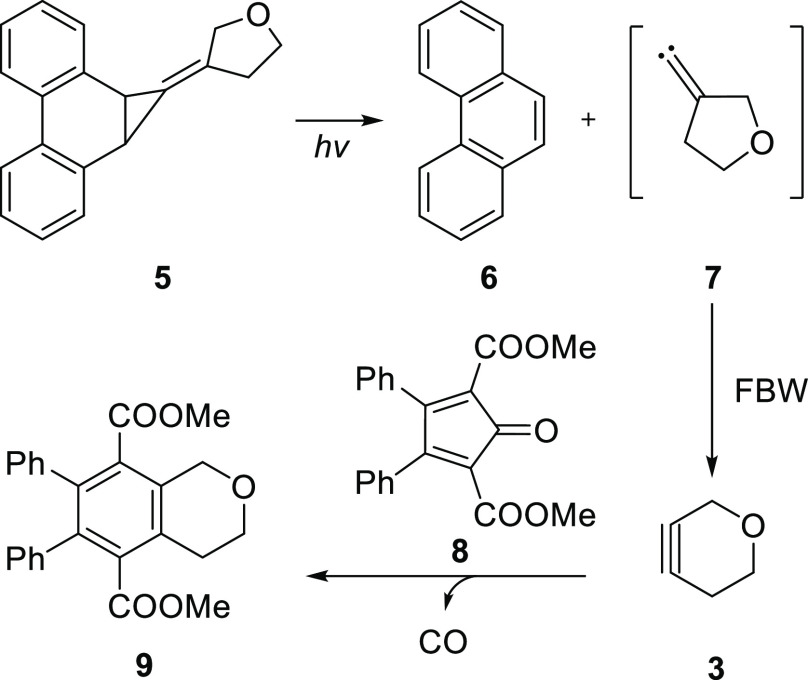
Photolytic Generation of Alkylidene Carbene **7** and Its Conversion to Oxacycloalkyne **3**

While the FBW rearrangement of alkylidene carbene **7** generates oxacyclohexyne **3** as the sole product, this 1,2-shift can proceed through two distinct pathways, depending on which one of the two allylic carbon atoms undergoes migration.^22^ Our previous calculations at the CCSD(T)/cc-pVTZ//B3LYP/6-31+G* level of theory predicted a preference for migration of the oxygen-bound allylic carbon atom,^15^ but experimental evidence to verify this prediction was unavailable until this study. Furthermore, although the relative migratory aptitudes of aromatic groups in the FBW rearrangements of alkylidene carbenes have received some attention,^22^ those of the alkyl groups have not been investigated sufficiently enough to establish an overall trend.^23,24^ Still less attention has been given to the effects of heteroatoms on the migratory aptitude of alkyl groups.^21^ Elucidation of the rearrangement pathway, or pathways, of heterocyclic alkylidene carbenes such as **7** would help clarify the effect of heteroatoms on the migratory aptitudes of alkyl groups undergoing FBW rearrangements.

Herein, we determine the migratory preferences in the FBW rearrangements of 3-oxacyclopentyl alkylidene carbene **7** as well as its sulfur analog, 3-thiacyclopentyl alkylidene carbene **10**, through the use of isotopically enriched carbenes 7* and 10* (Scheme 2). The rearrangements of ^13^C-labeled substrates revealed a preference for migration of the heteroatom-bound allylic carbon atom in both heterocyclic alkylidene carbenes investigated. The greater migratory aptitude of the heteroatom-bound allylic carbon atom is likely due to two factors: anomeric interactions involving the lone pair of the heteroatom and the antibonding orbital of the migrating carbon–carbon bond and a heteroatom-induced destabilization of the competing rearrangement pathway. Both effects increase in strength with a more electronegative heteroatom, resulting in a greater difference between the transition state energies of the two rearrangement pathways in 7* compared to 10* (*vide infra*).

**Scheme 2:**
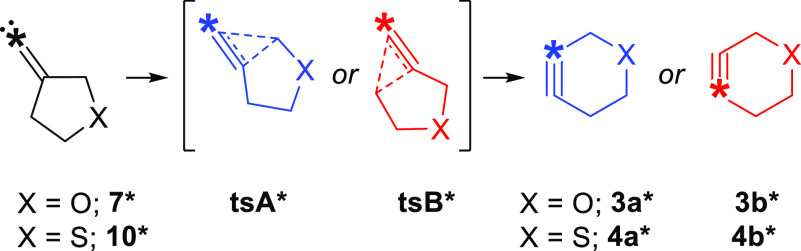
Isotopically Enriched Heterocyclic Alkylidene Carbenes Investigated in This Work *Denotes a ^13^C-enriched carbon atom.

Synthesis of ^13^C-labeled precursors 13* and 14* was performed in two steps (Scheme 3). First, cyclopropanation of phenanthrene (**6**) with 25% ^13^C-enriched chloroform gave 11*. A subsequent olefination reaction, developed by Takeda et al.,^25^ converted 11* into the desired compounds 13* and 14*.

**Scheme 3:**
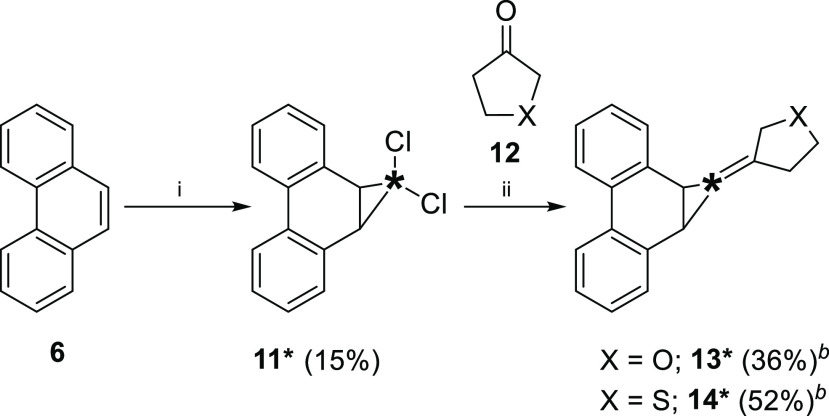
Synthesis of ^13^C-Labelled Alkylidene Carbene Precursors ∗ denotes a ^13^C-enriched carbon atom. Isolated yield. Reagents and conditions: (i) CHCl_3_, ^13^CHCl_3_, hexadecyltrimethylammonium chloride, 50% w/v aq. NaOH, reflux. (ii) Cp_2_TiCl_2_, Mg, P(OEt)_3_, **12**, 4 Å molecular sieves, THF.

Photolysis of ^13^C-labeled 3-oxacyclopentylphenanthrene derivative 13* in benzene (280–400 nm, 21 h) in the presence of cyclopentadienone **8** yielded adduct 9* as a mixture of two isotopomers, 9a* and 9b* (Scheme 4). The reaction likely proceeds through the release of alkylidene carbene 7*, which is isotopically enriched at the exocyclic carbene center.^16,26^ Subsequent FBW rearrangement of 7* generates oxacyclohexynes 3a* or 3b*, depending on which of the two allylic carbon atoms undergo migration. The strained alkynes add to diene **8**, generating 9a* and 9b* after the loss of carbon monoxide. Isotopomers 9a* and 9b* were observed in a ratio of 92:8,^27^ indicating that the migration of the oxygen-bound allylic carbon atom has a transition state energy that is 1.4 kcal/mol lower than that of migration of the alternative allylic carbon atom.^28^

**Scheme 4:**
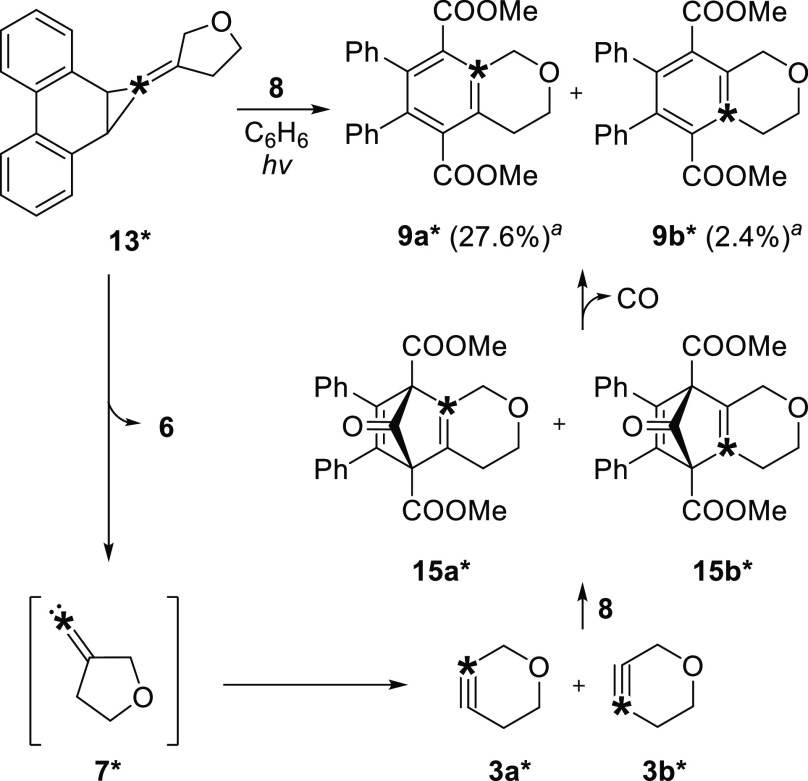
FBW Rearrangement of ^13^C-Labelled 3-Oxacyclopentyl Alkylidene Carbene 7^*^ Isotopomer yields determined via analysis of the ^13^C NMR spectrum of 9^*^.

The rearrangement of 3-thiacyclopentyl alkylidene carbene 10* also favored migration of the heteroatom-bound allylic carbon atom, albeit to a lesser extent. Photolysis of 3-thiacyclopentyl-phenathrene 14* in benzene (280–400 nm, 30 h) in the presence of **8** resulted in the formation of isotopomers 16a* and 16b* in a ratio of 61:39, corresponding to a difference in energy between the transition state barriers of the two rearrangement pathways of only 0.3 kcal/mol (Scheme 5).^28^ The low yield of 16* compared to that of its oxygen-containing counterpart 9* is likely not due to the failure of 14* to photolyze because despite its slower rate of photolysis 90% conversion of the starting precursor was ultimately achieved.

**Scheme 5:**
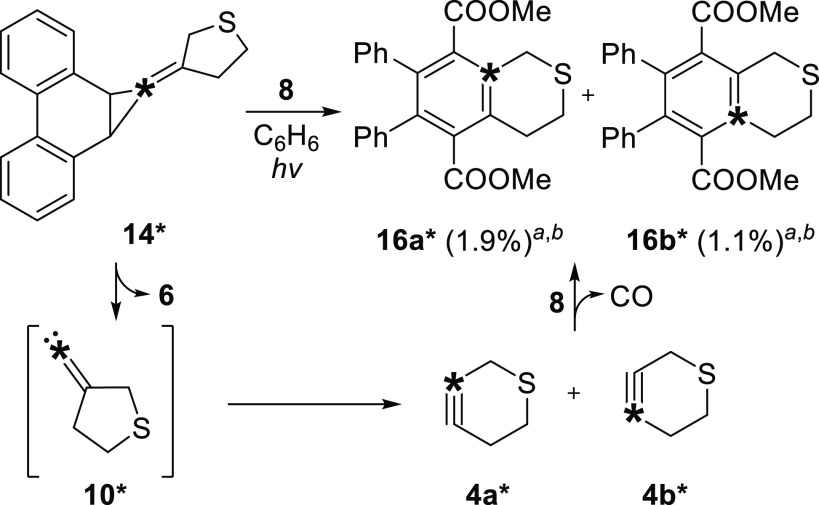
FBW Rearrangement of ^13^C-Labelled 3-Thiacyclopentyl Alkylidene Carbene 10^*^ Isotopomer yields determined via analysis of the C^13^ NMR spectrum of 16^*^.

The low yield of trapped product 16* following the photolysis of precursor 14* is likely due to the poor reactivity of the FBW rearrangement product, thiacyclohexyne 4*, with diene **8**. The reactivity of oxacyclohexyne **3** is enhanced by the presence of the oxygen atom in the propargylic position, which promotes distortion of the alkyne bond angles due to hyperconjugation between π_c≡c_ and σ*_c–o_ and lowers the energy required to distort the alkyne into the transition state geometry for cycloaddition (Figure 2).^29,30^ This effect is weaker in the case of propargylic sulfur atoms,^31^ resulting in less distortion of the alkyne bond angles. Ring-strain energy is moreover expected to be 4.6 kcal/mol^32^ lower in thiacyclohexyne **4** relative to oxacyclohexyne **3** due to the presence of the larger sulfur atom and longer carbon–sulfur bonds, resulting in a lower degree of strain-promoted reactivity (Figure 2).^6,33,34^

**Figure 2:**
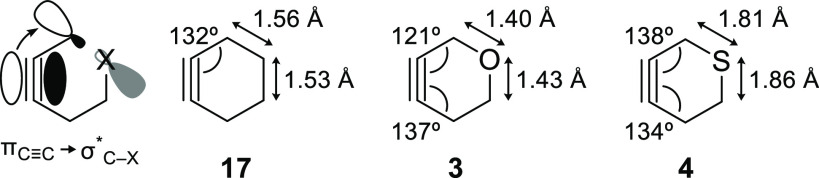
Effect of hyperconjugation on the bond lengths and alkynyl bond angles of heteroatomic cyclohexyne derivatives. Optimized structures were calculated at the PBE0/def2-TZVP level of theory.

The preference for migration of the heteroatom-bound, allylic carbon atom in the FBW rearrangements of alkylidene carbenes **7** and **10** likely arises through a hyperconjugative interaction between the lone pair of the heteroatom and the antibonding orbital of the migrating carbon–carbon bond (Figure 3A, *n*_X_ → σ*_C–C_).^8^ In the case of oxacyclopentyl alkylidene carbene **7**, the shortening of the C–O bond adjacent to the migrating bond in the transition state of the major migratory pathway provides evidence of this effect (Figure 3B, **7-tsA**). There is no such evidence for hyperconjugation in the transition state of the rearrangement of thiacyclopentyl alkylidene carbene **10**, during which the length of the C–S bond remains unchanged from the ground-state conformation (**10-tsA**). The relatively long length of the C–S bond results in a weaker interaction (*n*_S_ → σ*_C–C_) in alkylidene carbene **10** compared to that in alkylidene carbene **7** (*n*_O_ → σ*_C–C_) and likewise a smaller preference for the major pathway of rearrangement.^35^ The potential for hyperconjugation is also lessened by the hybridization of the sulfur atom in **10**, in which the sigma-type lone pair electrons involved in the interaction occupy an orbital that contains relatively little p-character.^36^

**Figure 3:**
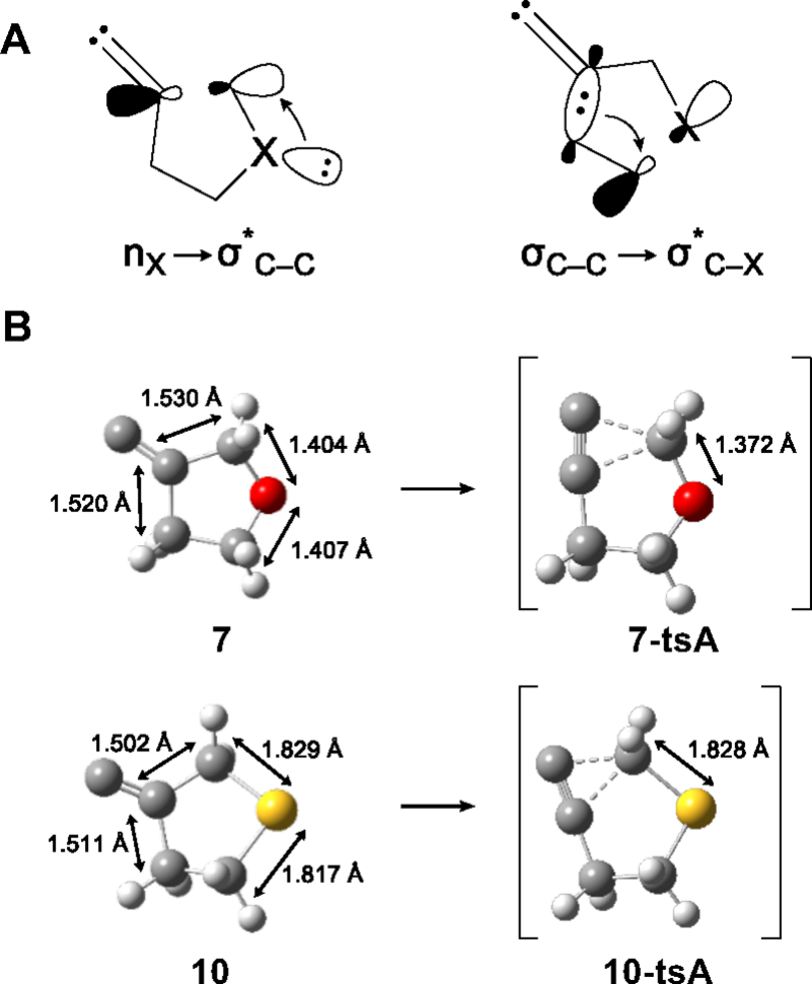
(A) Hyperconjugative effects in heterocyclic alkylidene carbenes. (B) Bond lengths within alkylidene carbenes **7** and **10** and within transition states **7-tsA** and **10-tsA**.

Hyperconjugative effects involving the heteroatom in alkylidene carbenes **7** and **10** may also inhibit the minor pathway of rearrangement (Scheme 2, **tsB**). Within the five-membered rings of **7** and **10**, the carbon–carbon bonds involved in the minor migratory pathways are well situated to donate into the σ*_C–X_ orbitals (Figure 3A, σ_C–C_ → σ*_C–X_), thereby inhibiting the migration of these bonds through FBW rearrangements.^37,38^

Computational experiments conducted using the ORCA program^39−41^ at the CCSD(T)/def2-TZVPP//PBE0/def2-TZVP^42−49^ level of theory agree with the experimental data, indicating that migration of the heteroatom-bound allylic carbon atom is favored in the FBW rearrangements of heterocyclic alkylidene carbenes **7** and **10** (Figure 4, Scheme 6). Consistent with the experimental data, the difference in the calculated transition state energies between the two rearrangement pathways is larger in the 3-oxacyclopentyl alkylidene carbene **7** (ΔΔ*G*^‡^ = 3.61 kcal/mol) than in the 3-thiacyclopentyl alkylidene carbene **11** (ΔΔ*G*^‡^ = 1.39 kcal/mol).

**Table 1:** 

Carbene	TS	Δ*G*^‡^ theoretical	tsB-tsA theoretical (experimental)
**7**	tsA	9.90 kcal/mol	3.61 (1.4) kcal/mol
	tsB	13.51 kcal/mol	
**10**	tsA	10.71 kcal/mol	1.39 (0.3) kcal/mol
	tsB	12.10 kcal/mol	

**Figure 4:**
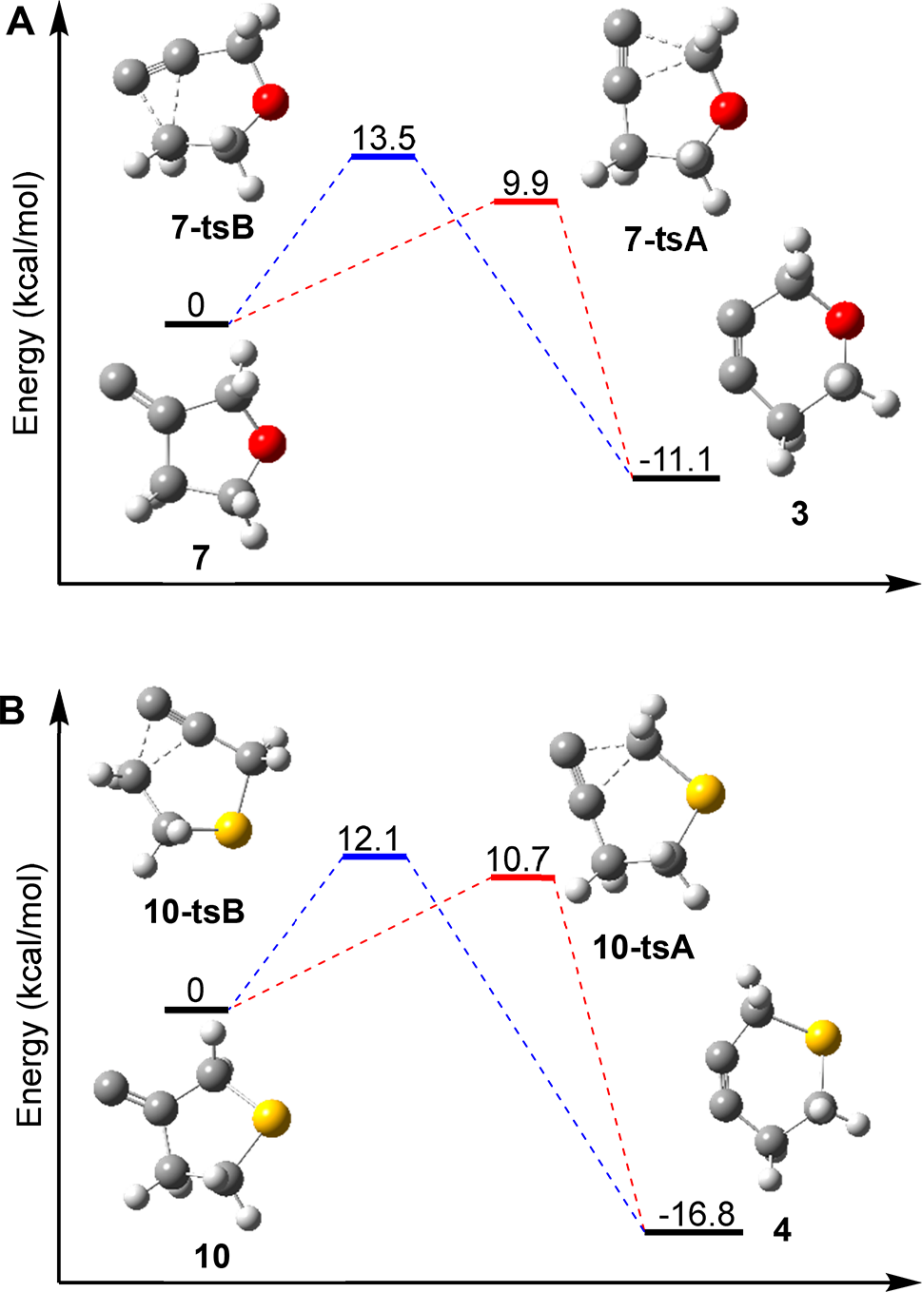
Transition state energies in the FBW rearrangements of heterocyclic alkylidene carbenes **7** (A) and **10** (B), computed at CCSD(T)/def2-TZVPP//PBE0/def2-TZVP.

**Scheme 6:**
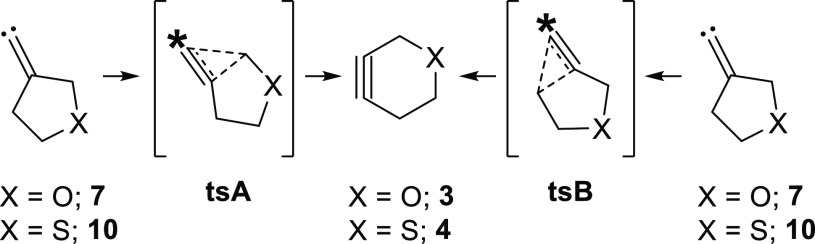
Experimentally and Theoretically Calculated Differences in Transition State Energies between the Two FBW Rearrangement Pathways in Heterocyclic Alkylidene Carbenes **7** and **10**

In addition to promoting migration of the heteroatom-bound allylic carbon through an anomeric interaction, the oxygen atom in alkylidene carbene **7** likely also has an inductively destabilizing effect on the competing migratory pathway. Conversion of the alkylidene moiety to an alkyne involves a transition state in which the endocyclic β-carbon atom is sp-hybridized, while the lone pair electrons of the carbene are held in an sp^2^-like orbital on the exocyclic α-carbon (Scheme 6).^50^ The vacant p-orbital on C-β is weakly engaged with the lone pair electrons on both the migrating carbon atom and C-α, but in this underdeveloped state the incipient alkyne exhibits an electron deficiency at the β-carbon.^21,50,51^ Accordingly, an electron-donating group adjacent to the β-carbon can be expected to stabilize the transition state,^21^ while an electropositive group, such as the methylene sandwiched between C-β and oxygen (in **7-tsB**), would have a destabilizing effect (Scheme 7). This effect should be negligible in the case of 3-thiacyclopentyl alkylidene carbene **10** because the electronegativities of the carbon and sulfur atoms are effectively equal.

**Scheme 7:**
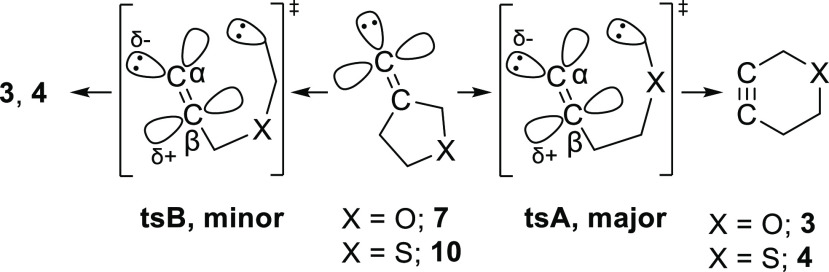
Electronic Localization in the FBW Transition State of Alkylidene Carbenes **7** and **10**

We have applied our previously developed methodology to generate ^13^C-labeled heterocyclic alkylidene carbenes 7* and 10*, which contain an oxygen and sulfur atom in the 3-position of the ring,^16^ respectively, and have determined the pathways of FBW rearrangement in these carbenes. While both possible pathways of migration occur in the rearrangements of both alkylidene carbenes, migration of the heteroatom-bound allylic carbon atom is preferred. 3-Oxacyclopentyl alkylidene carbene **7** exhibits a sizable difference in energy between the transition state barriers of the two rearrangement pathways, which likely arises from an anomeric interaction between the lone pair electrons of the oxygen atom and the antibonding orbital of the migrating bond.^8^ Computational experiments investigating bond lengths and localization of the lone pair electrons of the oxygen atom are consistent with this effect. Destabilization at the electropositive migratory terminus within the transition state of the competing rearrangement pathway may act to reinforce the preference for the major rearrangement pathway.^21^ These data provide valuable insights into the effect of heteroatoms on the migratory aptitudes of alkyl groups in FBW rearrangements, an area of study that is not well understood.

## Data Availability

The data underlying this study are available in the published article and its Supporting Information

## Supporting Information Available

Experimental procedures, NMR data, and computational details (PDF)
