# Supplementary material for: Formation of 3-Oxa- and 3-Thiacyclohexyne from Ring Expansion of Heterocyclic Alkylidene Carbenes: A Mechanistic Study
Source: Org Lett. 2023 Mar 1;25(9):1364–9. doi: 10.1021/acs.orglett.3c00042 (PMC10012261; doi:10.1021/acs.orglett.3c00042)
Supplement: Supplementary file 1 — ol3c00042_si_001.pdf [file ol3c00042_si_001.pdf]

Supporting Information

Formation of 3-Oxa- and 3-Thiacyclohexyne from Ring Expansion of Heterocyclic Alkylidene Carbenes: A Mechanistic Study

T. E. Anderson[a], Dasan M. Thamattoor*[a], and David Lee Phillips**[b]

[a] Department of Chemistry, Colby College, Waterville, ME 04901 (USA)

[b] Department of Chemistry, The University of Hong Kong, Pokfulam Road, Hong Kong S.A.R. 999077

*E-mail: dmthamat@colby.edu

**E-mail: phillips8hk@gmail.com

Table of Contents

General Notes 2

Computational Procedures 2

Energy Calculations 3

Synthetic Procedures 3

Experimental Spectra 7

Computational Data. 14

References 24

General Notes.

Tetrahydrofuran was degassed by purging with nitrogen, and dried by passage through two activated alumina columns (2 ft × 4 in). Other solvents and reagents were used as obtained from commercial sources. Medium pressure flash chromatography was performed on an automated system using prepacked silica gel columns (70−230 mesh) with the indicated eluents. Proton (^1H) and proton-decoupled carbon $^{13}C\{^1H\}$ NMR spectra were recorded in $CDCl_3$ at 500 and 126 MHz, respectively. The data are reported as follows: chemical shift in ppm referenced to residual solvent (^1H NMR: $CDCl_3$ δ 7.26; ^{13}C NMR: $CDCl_3$ δ 77.2, multiplicity, coupling constants (Hz), and integration. Structures were determined using COSY, HSQC, and HMBC experiments. Overlapping carbon peaks were identified using HSQC experiments and integration of ^{13}C NMR spectra. High resolution mass spectra (HRMS) data were obtained on an Agilent 6230 TOF Mass Spectrometer. Infrared spectra (resolution 4.0 cm^{-1}) were acquired on solid samples with an FTIR instrument equipped with an attenuated total reflectance (ATR) accessory. Photolysis experiments were conducted with a Newport 200 W Xe-Hg arc lamp (model # 6290; horizontal intensity 600 cd) with a Newport 280–400 dichroic mirror (model # 66245) fitted in a Newport 67005 Housing with a Newport 69907 Universal Arc Lamp Power Supply. All photolysis reactions were conducted in quartz glassware positioned 30 cm away from the light source. All reactions were performed under an atmosphere of argon in glassware that had been dried in an oven at 120 °C unless otherwise stated.

Computational Procedures.

All quantum chemical calculations were performed using Orca (version 5.0).[1-3] Geometries were optimized using hybrid density functional theory (PBE0)[4] using Ahrlich's def2-TZVP basis set.[5] Frequency calculations were performed to verify the nature of the stationary points as minima (0 imaginary frequency) or maxima (1 imaginary frequency). Single point energy calculations were performed using domain-based local pair natural orbital coupled-cluster [DLPNO-CCSD(T)][6-15] methods in combination with Ahrlich's def2-TZVPP basis set.[5] The auxiliary basis sets def2/J[16] and def2-TZVPP/C[17-18] were also used for density functional theory and coupled-cluster methods, respectively. All density functional theory calculations used Grimme's atom-pairwise dispersion correction with Becke–Johnson damping (D3BJ).[19-21] Acceleration of SCF and exchange integral calculations was accomplished by invoking the resolution-of-identity[22] option and the chain-of-spheres[23-25] algorithm, respectively (RIJCOSX). T1 diagnostic values[26] for coupled-cluster calculations were <0.02, indicating that the systems are adequately described by a single reference wave function. Transition state geometries were calculated using nudged elastic band with transition state optimization (NEB-TS).[27-29] Localized molecular orbital calculations were performed with the Gaussian 16 (Revision C.01)[30] computational package, using the Foster–Boys[31] localization method. GaussView (version 6.0)[32] and/or ChemCraft[33] were used to visualize computational data.

Energy Calculations.

Gibbs free energies were determined from the computational data by adding the electronic energy E_{el}, calculated at the DLPNO-CCSD(T) level, to the Gibbs free energy minus the electronic energy (G-E_{el}), calculated at the PBE0 level.[34] Differences in transition state energies ΔΔG‡ for the alkylidene carbene rearrangements, which were assumed to be irreversible, were derived from the experimental data based on the natural logs of the ratios of the two products.[35] The ratios of the two isotopomers were determined via integration of the purified ^{13}C NMR spectrum. These calculations were based on the assumption that the product distribution ratio of the two isotopomers reflects the ratio of the rate constants for their formation.

Strain energies of oxacyclohexyne **4** and thiacyclohexyne **3** were calculated using isodesmic equations similar to those described by Bach,[36] correcting for the nonzero strain energy of cyclohexane (2.2 kcal/mol, Scheme S1). Geometry optimization and energy calculations were performed at the PBE0/def2-TZVP level of theory.

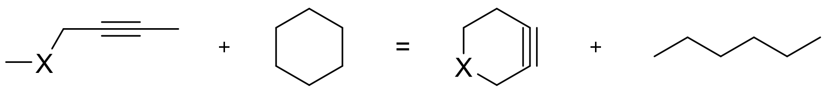

X = O, SE = 43.0 kcal/mol
X = S, SE = 38.4 kcal/mol

Scheme S1. Calculated Strain Energies (SEs) of cyclohexyne derivatives

Synthetic Procedures.

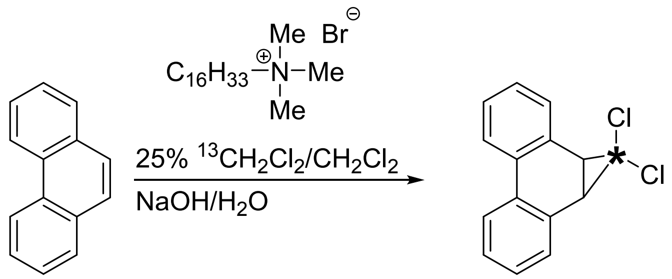

6 **11***

^{13}C-Labelled dichlorocyclopropyl phenanthrene 11*: To a pear-shaped round-bottom flask was added chloroform-^{13}C (7.50 mL, 83.8 mmol), chloroform (22.5 mL, 251 mmol), and phenanthrene (**6**, 26.0 g, 146 mmol). The flask was cooled to 0 °C in an ice bath, and hexadecyltrimethyl ammonium chloride (0.467 g, 1.46 mmol) was added. Aqueous sodium hydroxide (55 mL, 50% *w/v*) was then added via addition funnel over 15 min. The addition funnel was replaced with a dry

condenser, and the reaction mixture was heated at 50 °C in a heating mantle for 24 h. Water (350 mL) was then added to the reaction mixture, and the insoluble material was removed by filtration then purified by flash column chromatography (hexanes). The organic layer was separated from the filtrate and the solvent was removed *in vacuo*. The resulting solid was purified by recrystallization from hexanes. Purification yielded ^{13}C-labelled dichlorocyclopropyl phenanthrene (**11***) as a white, flakey solid (5.88 g, 15%). ^{1}H NMR (500 MHz, $CDCl_3$) δ 8.01 (d, *J* = 7.8 Hz, 2H), 7.48 (d, *J* = 7.4 Hz, 2H), 7.43–7.37 (m, 2H), 7.37–7.31 (m, 2H), 3.41 (s, 1H).^{13}C NMR (500 MHz, $CDCl_3$) δ 131.4, 131.1, 128.3, 128.2, 128.1, 123.2, 59.0 (C*), 36.6. HRMS (ESI) *m/z*: $[M + H]^+$ Calcd for $C_{15}H_{10}Cl_2$ 260.0160; Found 260.0172.

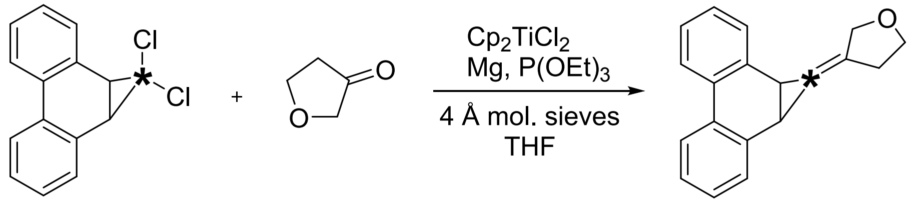

11* **12a** **13***

Precursor 13*: According to the procedure of Takeda *et al.*,[37] to a round-bottom flask charged with magnesium turnings (0.182 g, 7.50 mmol) and 4 Å molecular sieves (0.375 g) was added bis(cyclopentadienyl)titanium(IV) dichloride (1.87 g, 7.50 mmol) followed by anhydrous THF (14 mL). Triethyl phosphite (2.57 mL, 15.0 mmol) was added and the reaction mixture was stirred for 3 h, during which the color of the mixture turned from brown to dark green. A solution of ^{13}C-labelled dichlorocyclopropyl phenanthrene **11*** dissolved in THF (5 mL) was added and the reaction mixture was stirred for an additional 30 min. Dihydro-3(2H)-furanone (**12a**, 0.097 mL, 1.25 mmol) was then added, and the reaction mixture was stirred for an additional 16 h. Hexanes (45 mL) was added, and the resulting suspension was transferred to a plug of silica and eluted with hexanes (45 mL) followed by a solution of hexanes and ethyl acetate (10:90, 100 mL). The elution was concentrated *in vacuo*, and purification of the resulting residue by flash chromatography (0:100→15:85 ethyl acetate:hexanes) afforded precursor **13*** as a white solid (0.116 g, 36%). mp = 103–106 °C. ^{1}H NMR (500 MHz, $CDCl_3$) δ 8.00–7.92 (m, 2H), 7.42–7.37 (m, 1H), 7.37–7.32 (m, 1H), 7.28–7.23 (m, 4H), 4.32 (d, *J* = 12.9 Hz, 1H), 4.03 (d, *J* = 12.9 Hz, 1H), 3.89 (dt, *J* = 7.5, 7.0 Hz, 1H), 3.71 (dt, *J* = 7.6, 7.3 Hz, 1H), 3.16 (m, 2H), 2.63–2.53 (m, 1H), 2.41–2.30 (m, 1H). ^{13}C NMR (500 MHz, $CDCl_3$) δ 132.9, 132.8, 129.4, 129.3, 129.0, 128.9, 128.3, 128.0, 127.9, 126.33, 126.31, 123.52, 123.50, 115.6 (C*), 69.5, 68.8, 30.9, 22.3, 21.9. HRMS (ESI) *m/z*: $[M + H]^+$ Calcd for $C_{19}H_{16}O$ 260.1201; Found 260.1213.

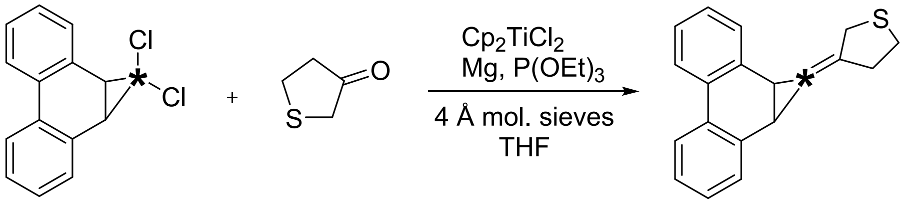

11* **12b** **14***

Precursor 14*: Following the procedure for the synthesis of precursor **13***, magnesium turnings (0.278 g, 11.45 mmol), 4 Å molecular sieves (0.573 g), bis(cyclopentadienyl)titanium(IV) dichloride (2.85 g, 11.45 mmol), triethyl phosphite (3.93 mL, 22.9 mmol), ^{13}C-labelled dichlorocyclopropyl phenanthrene **11***, and dihydro-3(2H)-thiophene (**12b**, 0.163 mL, 1.191 mmol) were combined in THF (30 mL) to afford precursor **14*** as a white solid (0.273 g, 52%): mp = 117–120 °C. ^{1}H NMR (500 MHz, $CDCl_3$) δ 8.01–7.92 (m, 2H), 7.41–7.33 (m, 2H), 7.29–7.22 (m, 4H), 3.51 (d, *J* = 13.9 Hz, 1H), 3.27 (d, *J* = 13.9 Hz, 1H), 3.22–3.15 (m, 2H), 2.86–2.76 (m, 1H), 2.74–2.62 (m, 2H), 2.55–2.44 (m, 1H). ^{13}C NMR (500 MHz, $CDCl_3$) δ 132.9, 132.7, 129.41, 129.35, 129.31, 128.94, 128.89, 128.0, 127.9, 126.4, 126.3, 123.54, 123.52, 118.2 (C*), 34.7, 33.6 , 31.0, 22.67, 22.65. IR (ATR) 2941, 1485, 1441, 1423, 1201, 959, 770, 730 cm^{-1}. HRMS (ESI) *m/z*: $[M + H]^+$ Calcd for $C_{19}H_{16}S$ 276.0973; found 276.0962.

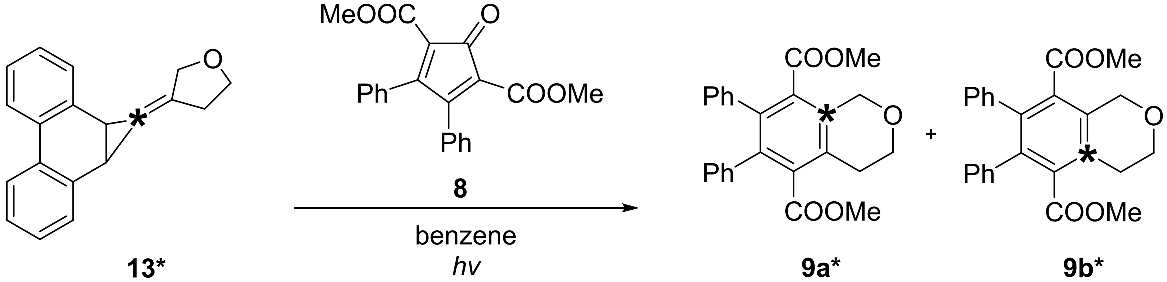

13* **9a*** **9b***

Photolysis of 13*: Precursor **13*** (0.114 g, 0.436 mmol) and 2-oxo-4,5-diphenyl-cyclopenta-3,5-diene-1,3-dicarboxylic acid dimethylester (**8**, 0.133 g, 0.383 mmol) were dissolved in benzene (5 mL) and transferred to an argon-flushed quartz cuvette. The reaction mixture was placed in front of a mercury lamp and irradiated for 21 h, then the benzene was removed *in vacuo*. Purification of the resulting residue by flash chromatography (0:100→5:95 ethyl acetate:hexanes) afforded adduct **9** as a pale yellow solid as a mixture of isotopomers **9a** and **9b** (0.047 g, 30%): mp = 142–145 °C. ^{1}H NMR (500 MHz, $CDCl_3$) δ 7.17–7.07 (m, 6H), 7.02–6.93 (m, 4H), 4.83 (s, 2H), 4.01 (t, *J* = 5.5 Hz, 2H), 3.47 (s, 3H), 3.43 (s, 3H), 2.90 (t, *J* = 5.5 Hz, 2H). ^{13}C NMR (500 MHz, $CDCl_3$) δ 169.0, 168.6, 138.1, 138.0, 137.6, 137.5, 136.0, 132.1, 132.0 (C*), 130.3 (C*), 130.0, 129.9, 127.6, 127.6, 127.14, 127.10, 66.5, 64.7, 52.11, 52.08, 26.3. IR (ATR) 2943, 2864, 1722, 1434, 1309, 1198, 1116 cm^{-1}. HRMS (ESI) *m/z*: $[M + H]^+$ Calcd for $C_{25}H_{22}O_5$ 402.1467; Found 402.1504.

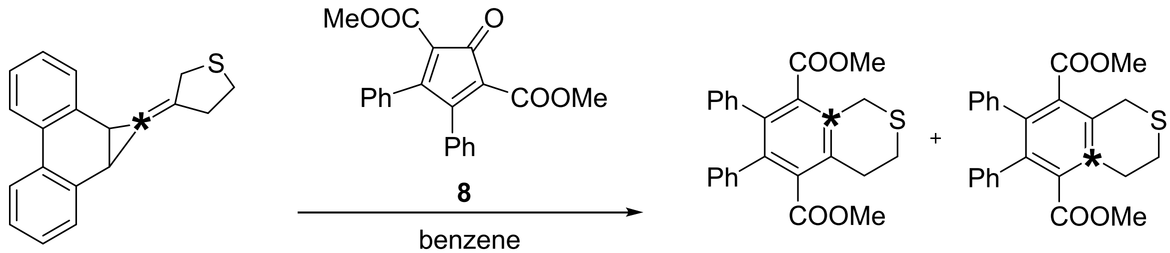

14* 16a* 16b*

Photolysis of 14*: Following the procedure for the photolysis of precursor **13***, precursor **14*** (0.271 g, 0.977 mmol) and 2-oxo-4,5-diphenyl-cyclopenta-3,5-diene-1,3-dicarboxylic acid dimethylester (**8**, 0.340 g, 0.977 mmol) were dissolved in benzene (15 mL) and irradiated for 30 h. Purification by flash chromatography (0:100 ethyl acetate:hexanes→5:95 ethyl acetate:hexanes) afforded adduct **16** as a pale solid as a mixture of isotopomers **16a*** and **16b*** (0.012 g, 3%): mp = 121–124 °C. ^{1}H NMR (500 MHz, $CDCl_3$) δ 7.14–7.08 (m, 6H), 7.02–6.97 (m, 4H), 3.77 (s, 2H), 3.46 (s, 6H), 3.06 (t, J = 5.6 Hz, 2H), 2.96 (t, J = 5.6 Hz, 2H). ^{13}C NMR (500 MHz, $CDCl_3$) δ 169.3, 169.1, 138.04, 137.99, 137.7, 137.3, 136.2, 134.4, 133.5 (C*), 132.2 (C*), 130.02, 130.01, 127.59, 127.58, 127.2, 127.1, 52.2, 52.1, 27.9, 26.8, 25.8. IR (ATR) 2950, 1723, 1435, 1312, 1206 cm^{-1}. HRMS (ESI) *m/z*: $[M + H]^+$ Calcd for $C_{25}H_{22}O_4S$ 418.1239; Found 419.1219.

Experimental Spectra.

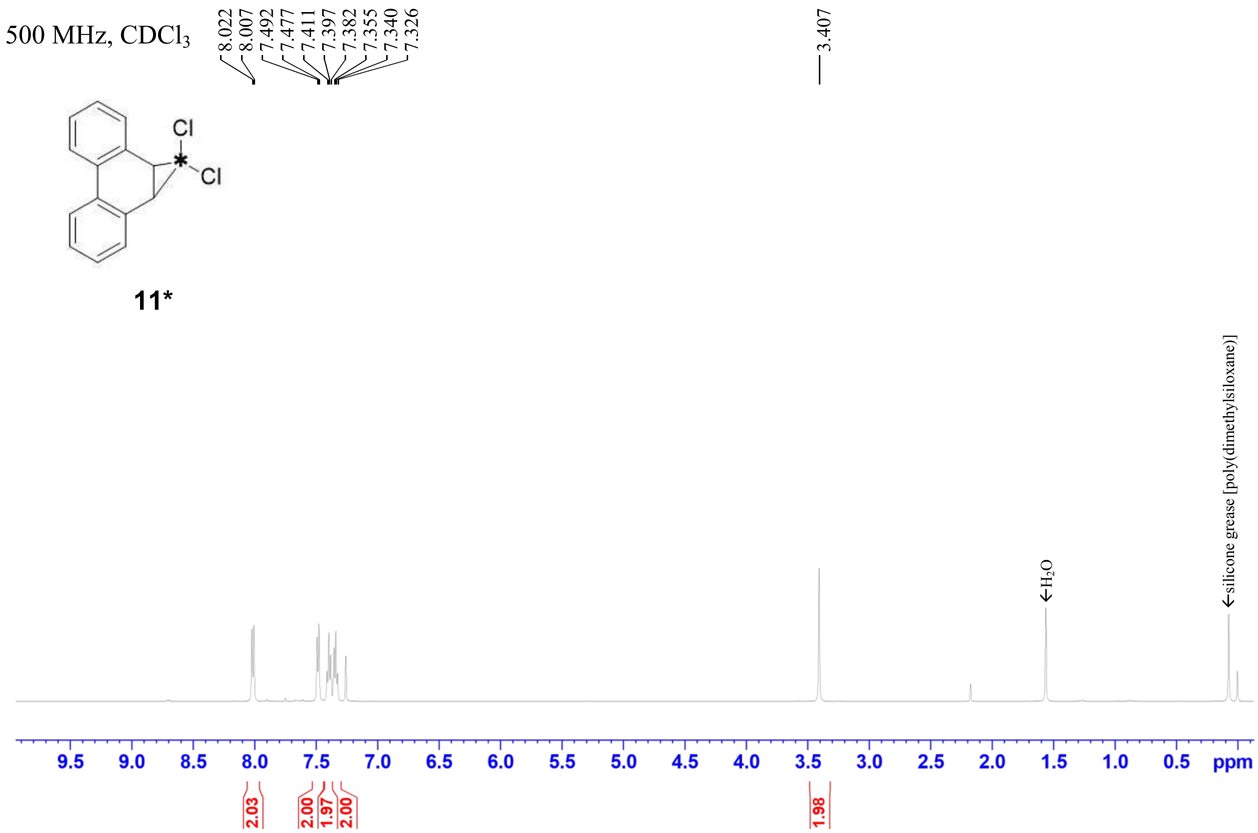

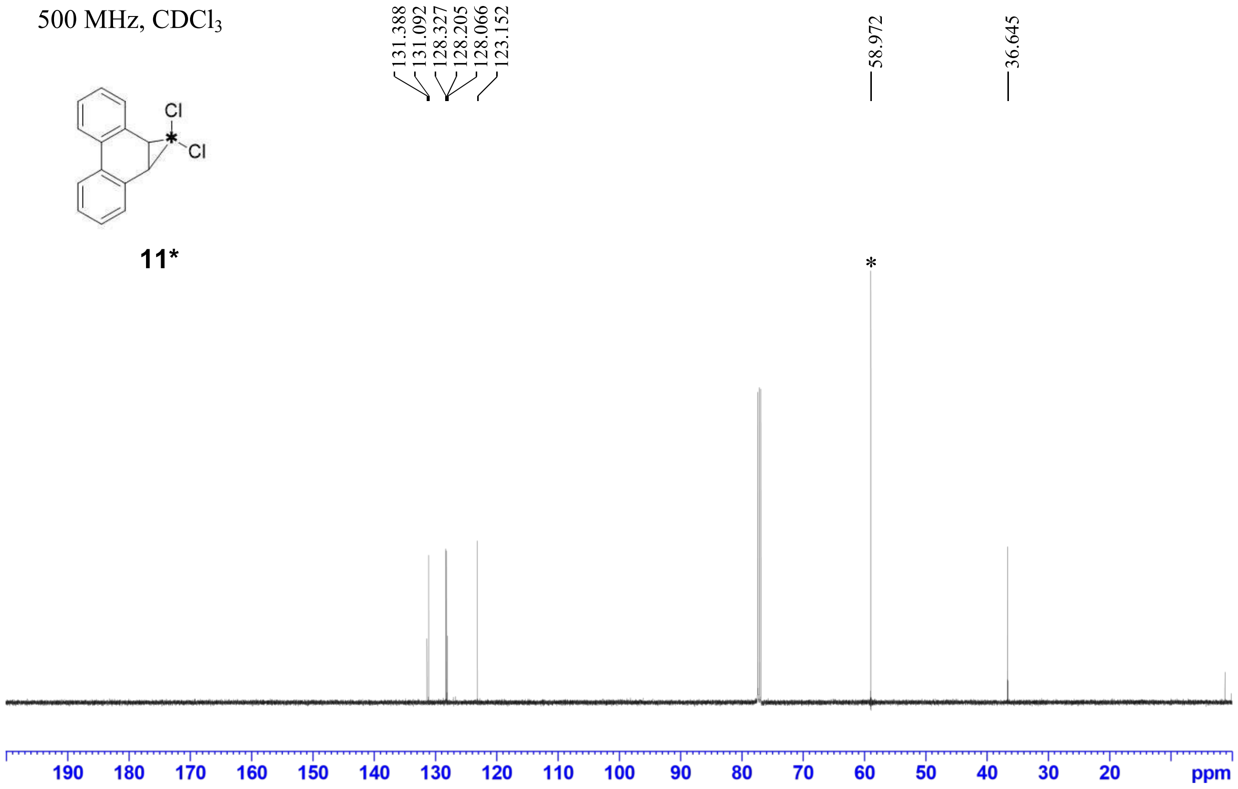

500 MHz, $CDCl_3$

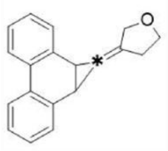

13*

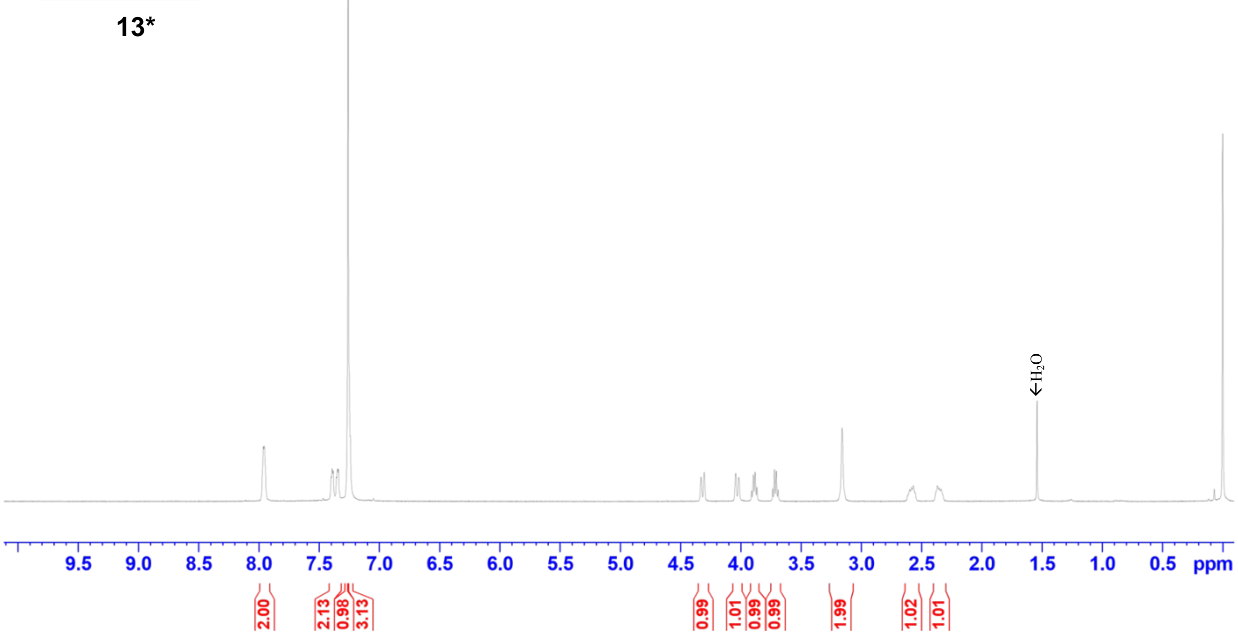

500 MHz, $CDCl_3$

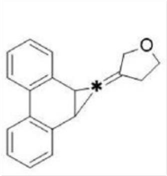

13*

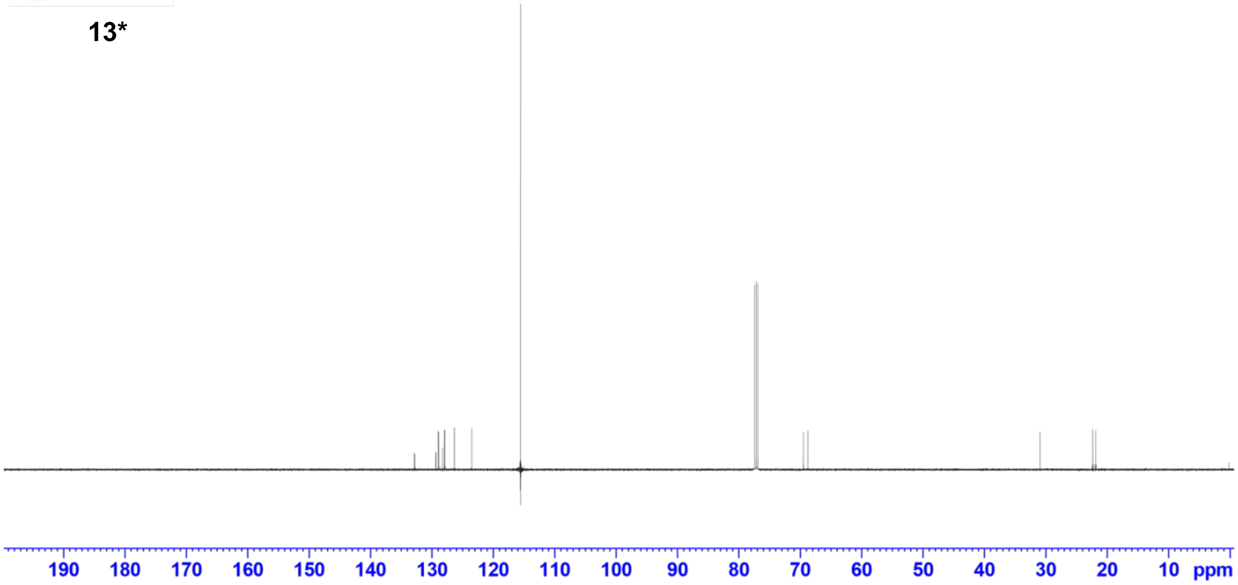

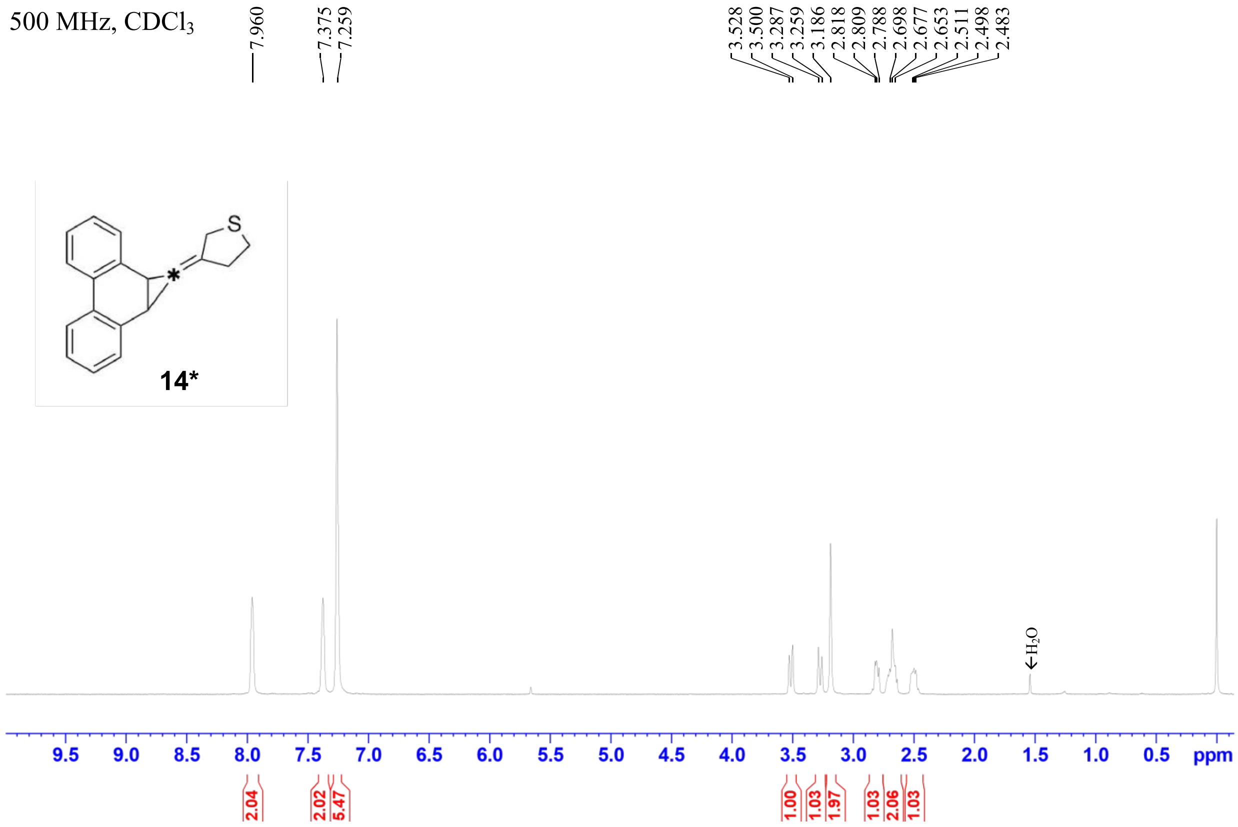
500 MHz, CDCl3
7.960
7.375
7.259
3.528
3.500
3.287
3.259
3.186
2.818
2.809
2.788
2.698
2.677
2.653
2.511
2.498
2.483
S
14*
H2O
9.5 9.0 8.5 8.0 7.5 7.0 6.5 6.0 5.5 5.0 4.5 4.0 3.5 3.0 2.5 2.0 1.5 1.0 0.5 ppm
2.04
2.02
5.47
1.00
1.03
1.97
1.03
2.06
1.03

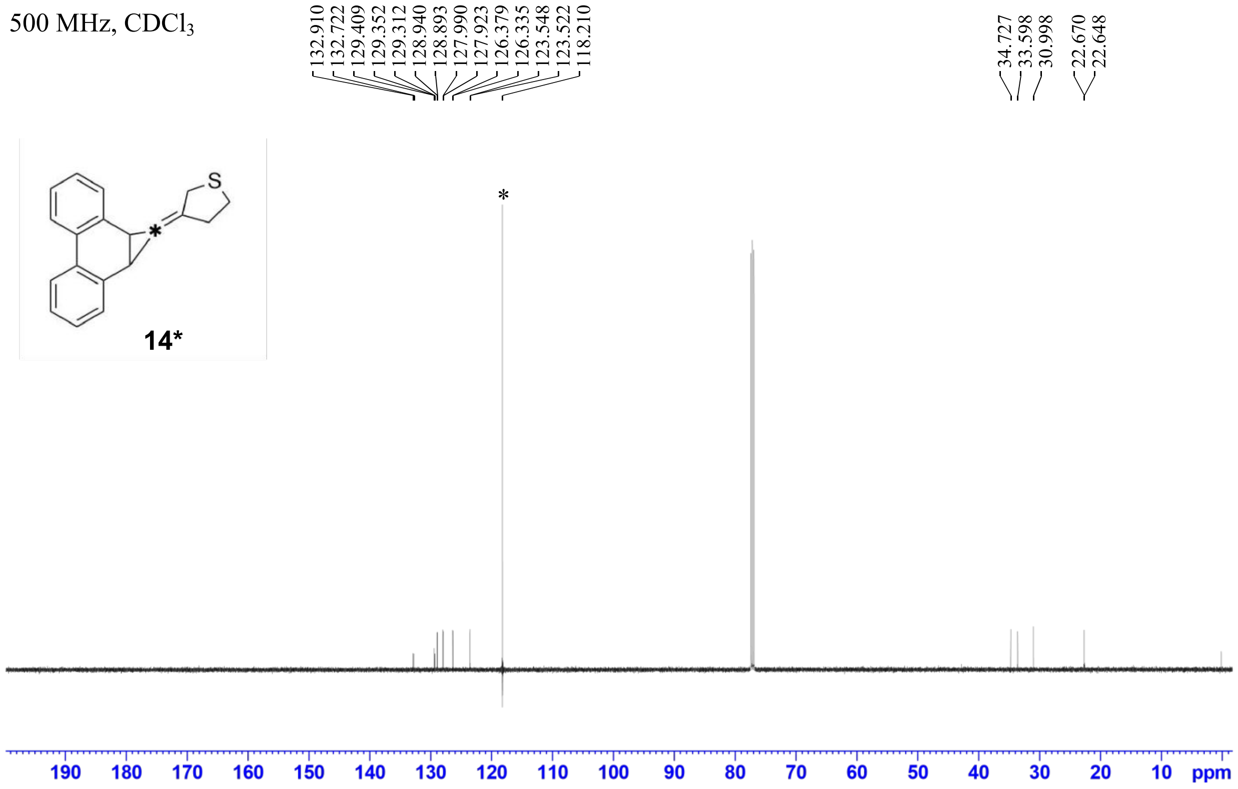
500 MHz, CDCl3
132.910
132.722
129.409
129.352
129.312
128.940
128.893
127.990
127.923
126.379
126.335
123.548
123.522
118.210
34.727
33.598
30.998
22.670
22.648
S
14*
*
190 180 170 160 150 140 130 120 110 100 90 80 70 60 50 40 30 20 10 ppm

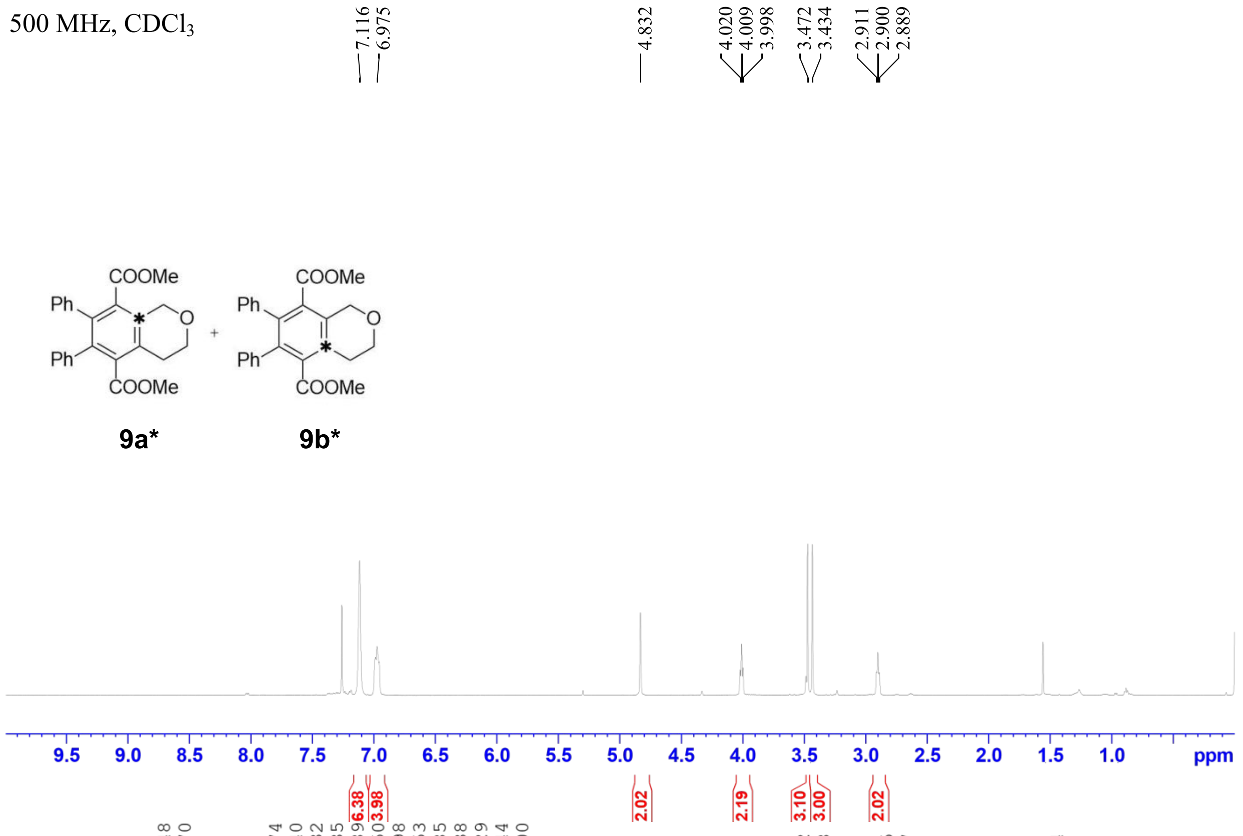

500 MHz, $CDCl_3$
7.116
6.975
4.832
4.020
4.009
3.998
3.472
3.434
2.911
2.900
2.889
COOMe
Ph
Ph
O
COOMe
+
9a*
9b*
9.5 9.0 8.5 8.0 7.5 7.0 6.5 6.0 5.5 5.0 4.5 4.0 3.5 3.0 2.5 2.0 1.5 1.0 ppm
6.38
3.98
2.02
2.19
3.10
3.00
2.02

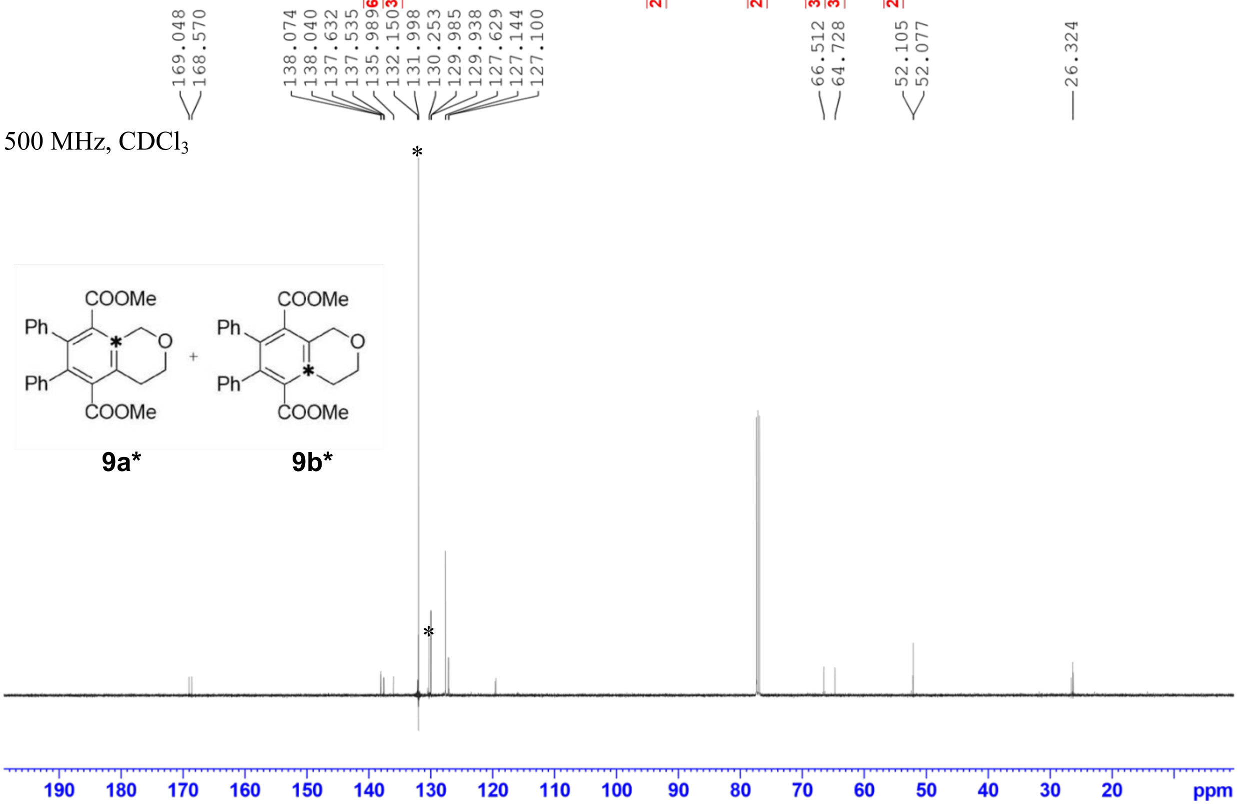

169.048
168.570
138.074
138.040
137.632
137.535
135.989
132.150
131.998
130.253
129.985
129.938
127.629
127.144
127.100
66.512
64.728
52.105
52.077
26.324
500 MHz, $CDCl_3$
*
*
COOMe
Ph
Ph
O
COOMe
+
9a*
9b*
190 180 170 160 150 140 130 120 110 100 90 80 70 60 50 40 30 20 ppm

HSQC

500 MHz, $CDCl_3$

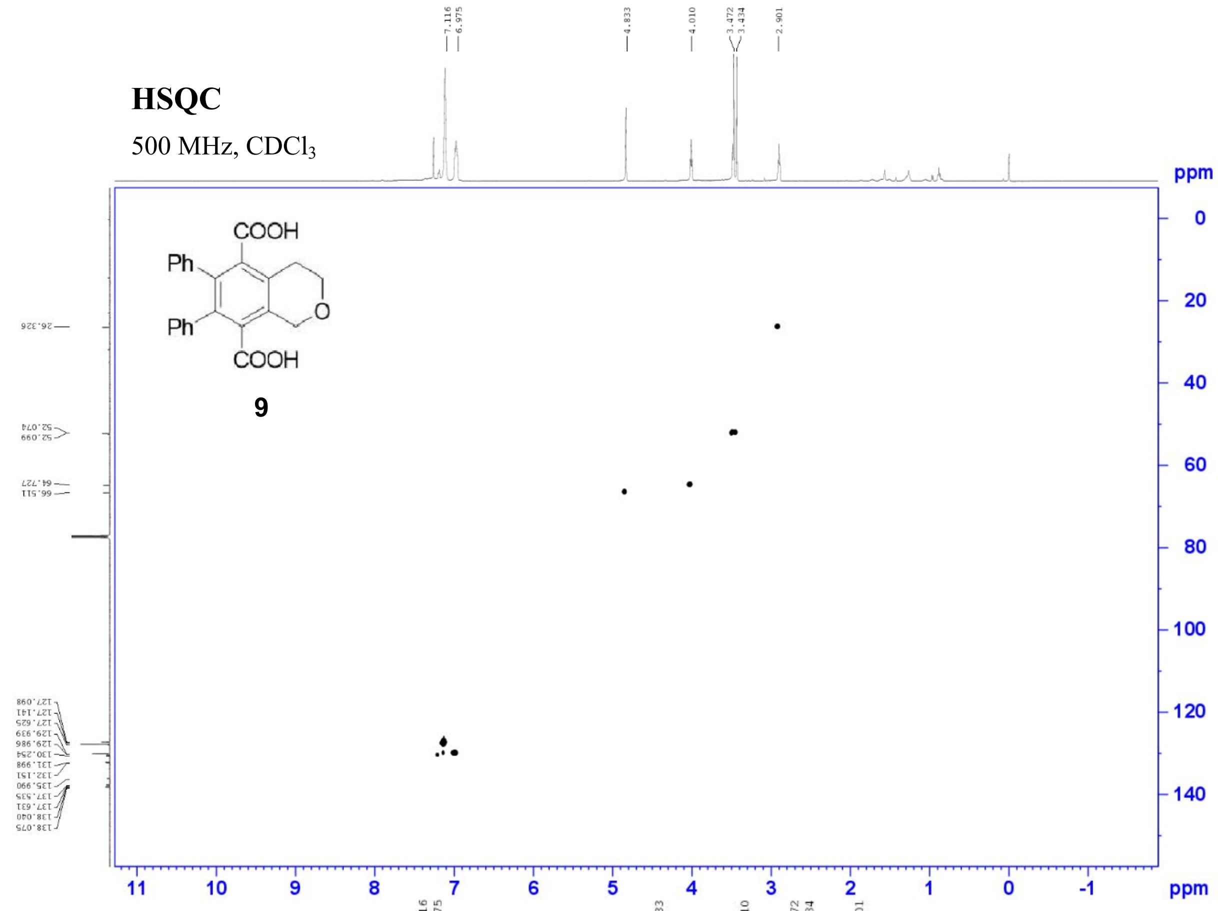

HMBC

500 MHz, $CDCl_3$

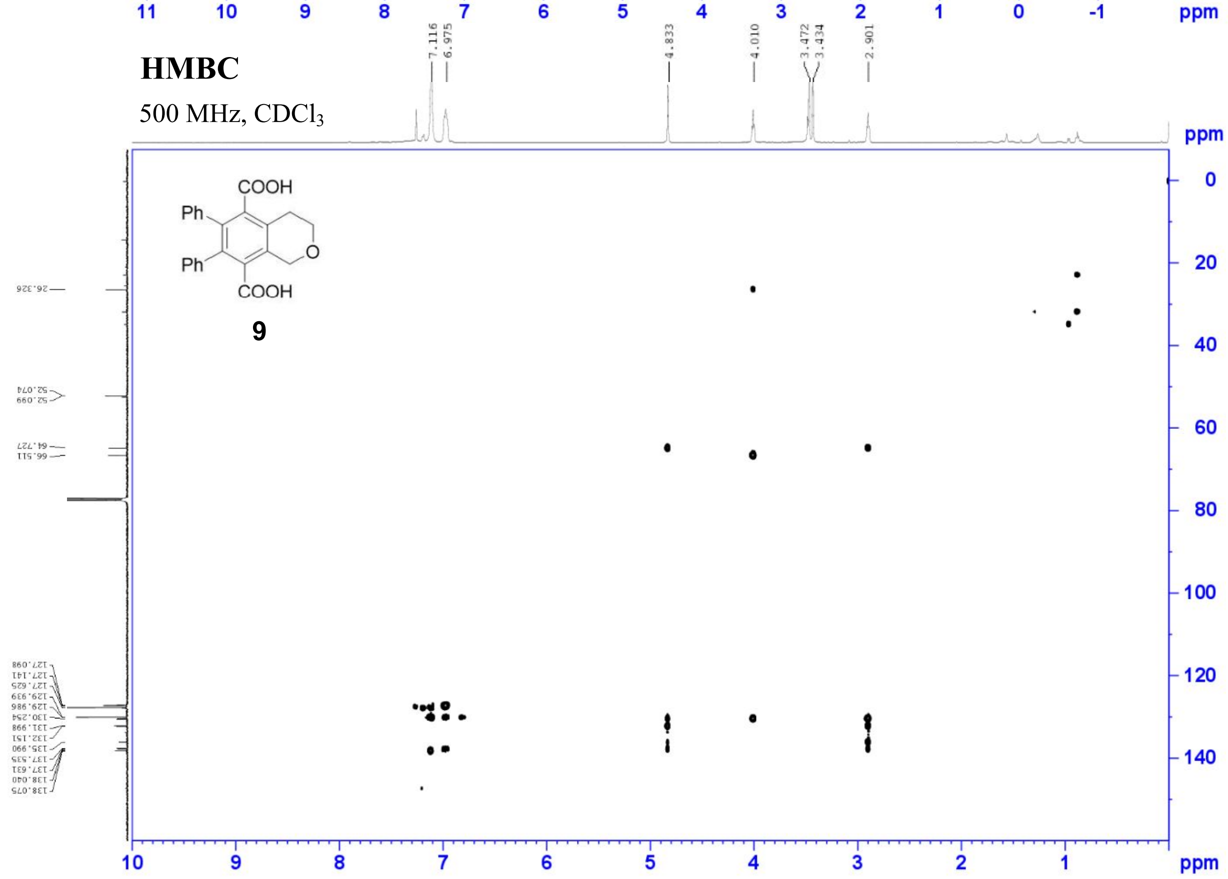

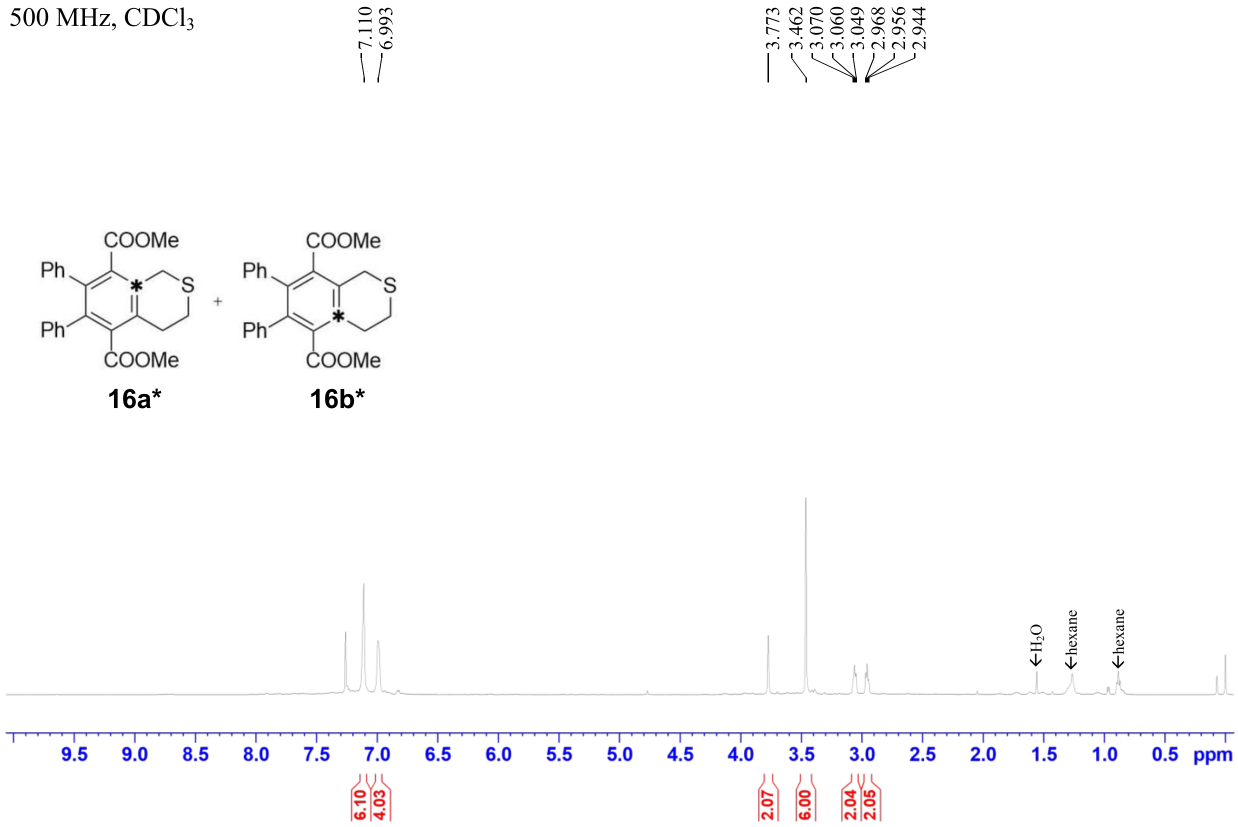
500 MHz, CDCl3
7.110
6.993
3.773
3.462
3.070
3.060
3.049
2.968
2.956
2.944
COOMe
Ph
Ph
S
COOMe
16a*
16b*
H2O
hexane
hexane
ppm
6.10
4.03
2.07
6.00
2.04
2.05

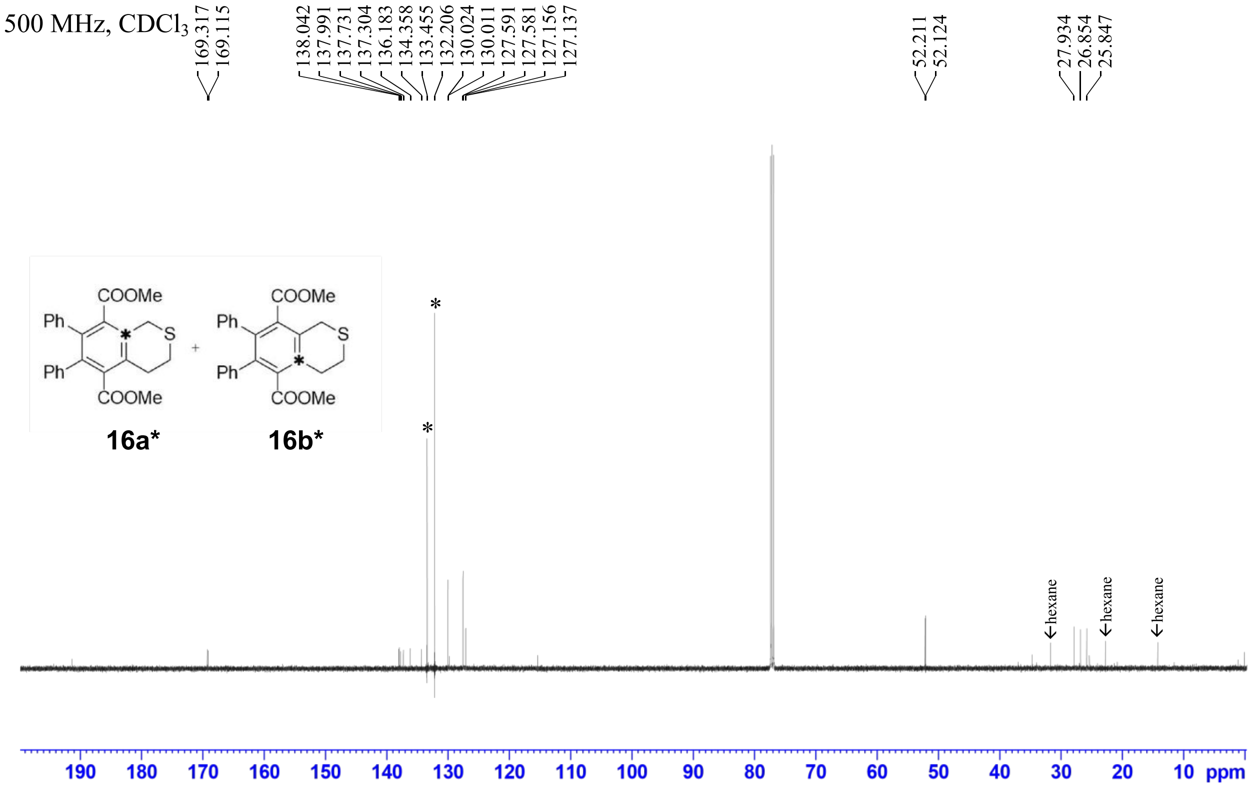
500 MHz, CDCl3
169.317
169.115
138.042
137.991
137.731
137.304
136.183
134.358
133.455
132.206
130.024
130.011
127.591
127.581
127.156
127.137
52.211
52.124
27.934
26.854
25.847
COOMe
Ph
Ph
S
COOMe
16a*
16b*
hexane
hexane
hexane
ppm

HSQC

500 MHz, $CDCl_3$

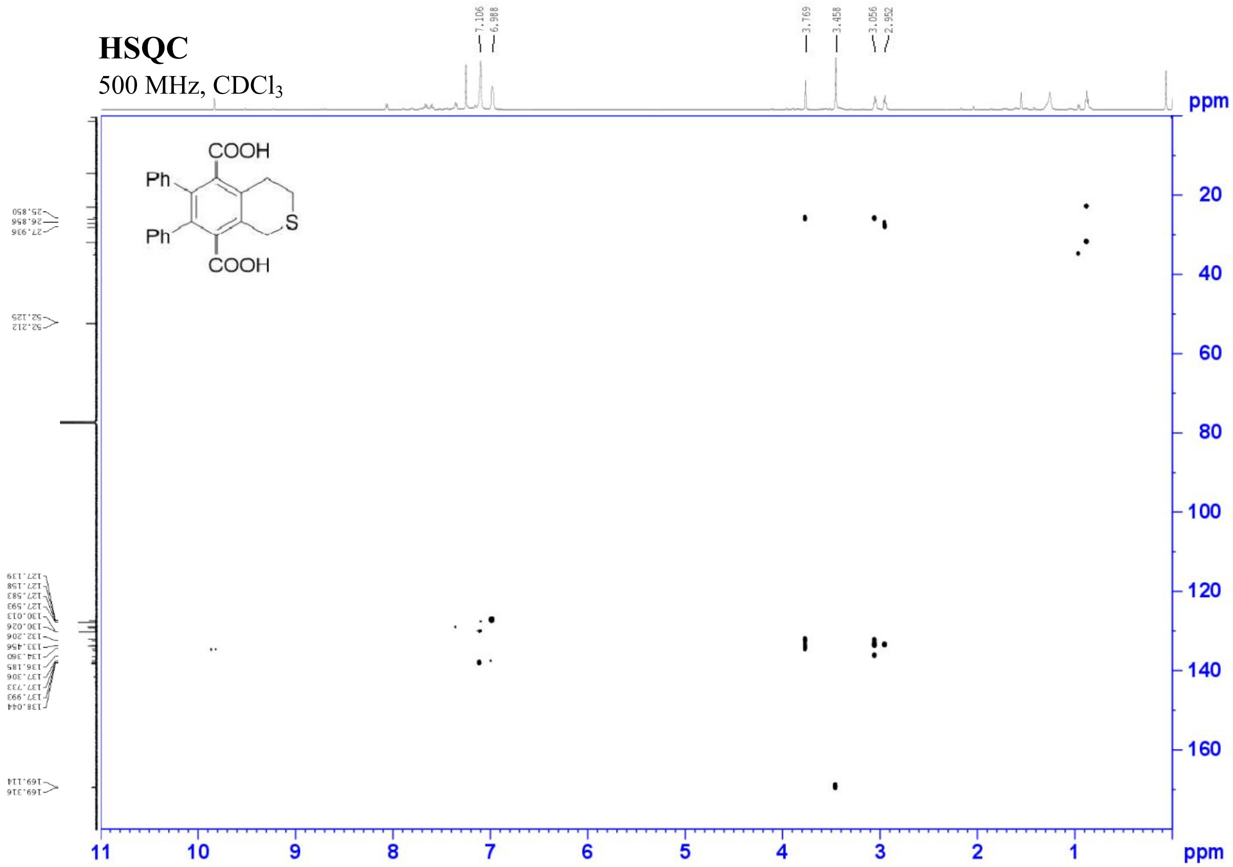

HMBC

500 MHz, $CDCl_3$

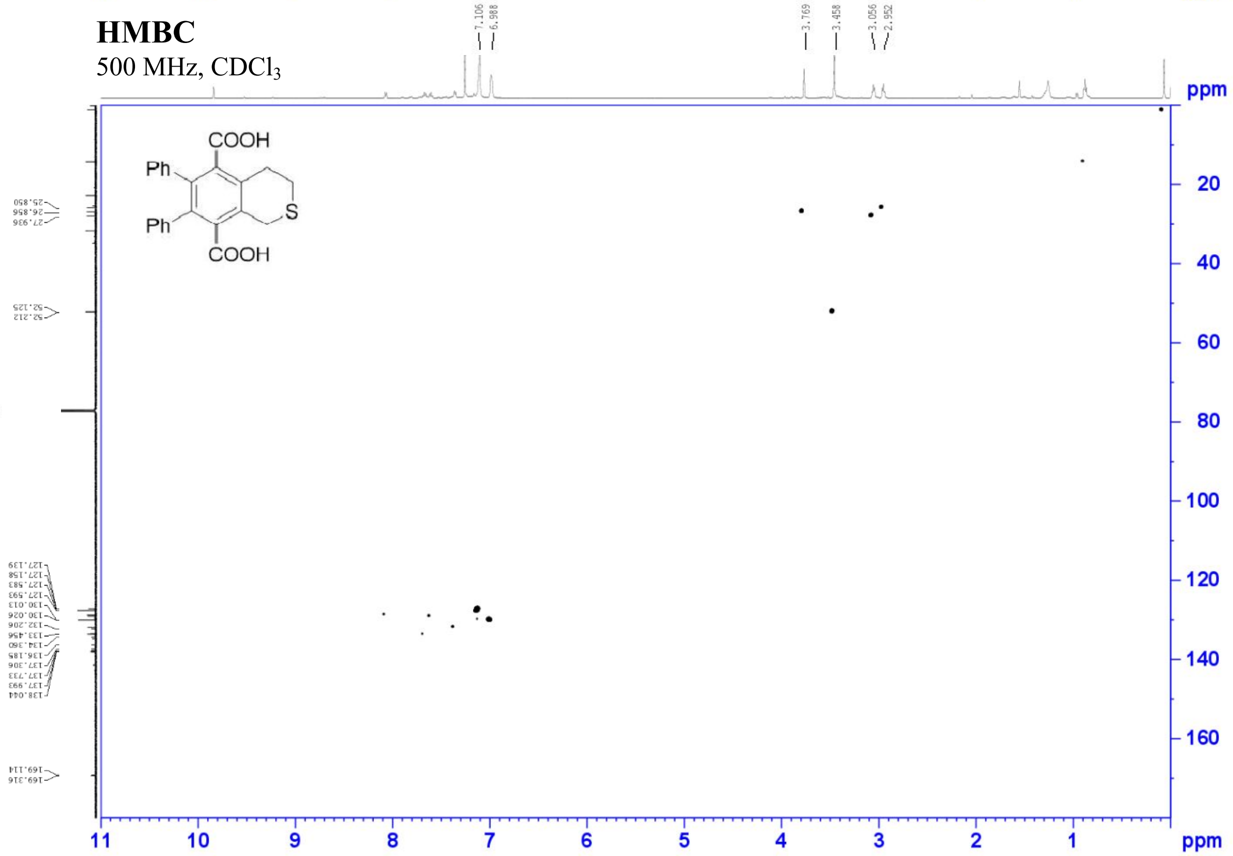

Computational Data.

Coordinates and energies for CCSD(T)/def2-TZVPP//PBE0/def2-TZVP optimized structures (at 298 K)

3-oxacyclopentyl alkylidene carbene 7, singlet

Charge = 0 Multiplicity = 1

C	0.005438000000	-0.033971000000	-0.136789000000
C	1.524506000000	-0.004562000000	-0.082941000000
C	1.931857000000	1.468947000000	-0.143011000000
O	0.742672000000	2.165017000000	0.125421000000
C	-0.292496000000	1.429400000000	-0.481296000000
H	-1.243659000000	1.782223000000	-0.082833000000
H	-0.281957000000	1.571799000000	-1.570987000000
H	2.303065000000	1.695378000000	-1.151268000000
H	2.682043000000	1.748260000000	0.594967000000
C	2.299291000000	-1.033982000000	0.012667000000
H	-0.385526000000	-0.736921000000	-0.871666000000
H	-0.392184000000	-0.290725000000	0.846581000000

G–E(el) = 0.06787815 Hartree
Final single point energy E(CCSD(T)) = -268.747632665602 Hartree

3-oxacyclohexyne (3), singlet

Charge = 0 Multiplicity = 1

C	-0.491641000000	0.026188000000	-0.304686000000
C	0.785419000000	-0.627879000000	-0.489424000000
C	2.363136000000	1.067740000000	-0.056698000000
O	1.195282000000	1.737546000000	0.428164000000
C	0.018707000000	1.523300000000	-0.292856000000
H	-0.739264000000	2.147383000000	0.184113000000
H	0.139096000000	1.831195000000	-1.339882000000
H	2.752598000000	1.567761000000	-0.951853000000
H	3.099037000000	1.147689000000	0.742905000000
C	1.957084000000	-0.321436000000	-0.384070000000
H	-1.215407000000	-0.089141000000	-1.114545000000
H	-0.970999000000	-0.249483000000	0.637679000000

G–E(el) = 0.06984526 Hartree
Final single point energy E(CCSD(T)) = -268.767354582517 Hartree

FBW rearrangement of 7, transition state A (7-tsA)

Charge = 0 Multiplicity = 1

C	-0.888059000000	-0.932820000000	0.113501000000
C	0.562379000000	-1.135553000000	0.173630000000
C	1.308879000000	0.625092000000	0.096587000000

O	0.096493000000	1.180252000000	0.420385000000
C	-0.982117000000	0.572644000000	-0.212216000000
H	-1.896444000000	1.024535000000	0.161703000000
H	-0.930694000000	0.725079000000	-1.294290000000
H	1.598982000000	0.824949000000	-0.931600000000
H	2.044853000000	0.981216000000	0.801559000000
C	1.798031000000	-1.168059000000	0.253956000000
H	-1.359319000000	-1.541934000000	-0.657220000000
H	-1.352983000000	-1.155400000000	1.074006000000

G–E(el) = 0.06793430 Hartree
Final single point energy E(CCSD(T)) = -268.731913007203 Hartree

FBW rearrangement of 7, transition state B (7-tsB)

Charge = 0 Multiplicity = 1

C	-0.757455000000	-0.994808000000	0.161426000000
C	1.143042000000	-0.659847000000	0.061760000000
C	1.307637000000	0.812923000000	0.099993000000
O	0.021519000000	1.289768000000	0.358328000000
C	-0.936919000000	0.472360000000	-0.241235000000
H	-1.915428000000	0.819782000000	0.084385000000
H	-0.885682000000	0.563300000000	-1.330970000000
H	1.693494000000	1.196046000000	-0.848477000000
H	1.970236000000	1.127250000000	0.903127000000
C	0.769312000000	-1.839390000000	0.085517000000
H	-1.332231000000	-1.616542000000	-0.517609000000
H	-1.077525000000	-1.170843000000	1.183755000000

G–E(el) = 0.06752996 Hartree
Final single point energy E(CCSD(T)) = -268.725762495984 Hartree

3-thiacyclopetnyl alkylidene carbene 10, singlet

Charge = 0 Multiplicity = 1

C	-0.003768000000	0.560298000000	-0.056267000000
C	0.335039000000	2.041080000000	0.041429000000
C	-0.843700000000	2.731212000000	-0.604886000000
C	-2.149585000000	2.166214000000	-0.124700000000
H	0.231111000000	0.171878000000	-1.047919000000
H	0.532537000000	-0.025825000000	0.691587000000
H	0.391938000000	2.357349000000	1.087122000000
H	1.282474000000	2.279682000000	-0.444213000000
H	-2.470200000000	2.672292000000	0.787911000000
H	-2.938190000000	2.243406000000	-0.872193000000
C	-0.686174000000	3.598120000000	-1.555115000000
S	-1.789000000000	0.411993000000	0.246550000000

G–E(el) = 0.06297473 Hartree

Final single point energy E(CCSD(T)) = -591.367964635456 Hartree

3-thiacyclohexyne (4), singlet

Charge = 0 Multiplicity = 1

C	-0.327485000000	0.638325000000	-0.012155000000
C	0.748604000000	1.715469000000	0.331600000000
C	0.168162000000	2.928103000000	-0.236304000000
C	-2.263313000000	2.648906000000	-0.667266000000
H	-0.215815000000	0.356623000000	-1.060956000000
H	-0.174690000000	-0.256751000000	0.598621000000
H	0.883889000000	1.816241000000	1.411437000000
H	1.704171000000	1.407390000000	-0.100476000000
H	-3.075453000000	3.184112000000	-0.172846000000
H	-2.551939000000	2.434862000000	-1.697533000000
C	-0.952278000000	3.255281000000	-0.556761000000
S	-2.051371000000	1.079139000000	0.311945000000

G–E(el) = 0.06517802 Hartree
Final single point energy E(CCSD(T)) = -591.396878098604 Hartree

FBW rearrangement of 10, transition state A (7-tsA)

C	0.621350000000	-1.165806000000	0.164834000000
C	1.263371000000	0.227412000000	0.177267000000
C	0.204698000000	1.161277000000	-0.242277000000
C	-1.553886000000	0.451284000000	-0.170978000000
H	0.562761000000	-1.555883000000	-0.850779000000
H	1.172503000000	-1.877990000000	0.776142000000
H	1.615507000000	0.476552000000	1.178126000000
H	2.106433000000	0.283160000000	-0.512891000000
H	-2.355835000000	0.919831000000	0.386073000000
H	-1.911170000000	0.111353000000	-1.135755000000
C	-0.682964000000	1.940160000000	-0.626429000000
S	-1.042767000000	-0.971350000000	0.856667000000

G–E(el) = 0.06280444 Hartree
Final single point energy E(CCSD(T)) = -591.350716000596 Hartree

FBW rearrangement of 10, transition state B (7-tsB)

C	0.594852000000	-1.074797000000	0.106283000000
C	1.206265000000	0.324747000000	0.089210000000
C	-0.467911000000	1.126256000000	-0.362084000000
C	-1.646341000000	0.308430000000	-0.045066000000
H	0.489170000000	-1.459737000000	-0.907634000000
H	1.258755000000	-1.744677000000	0.653470000000
H	1.501002000000	0.640791000000	1.084084000000
H	2.068669000000	0.313840000000	-0.567840000000

H	-2.355637000000	0.864545000000	0.565052000000
H	-2.146414000000	-0.040989000000	-0.947300000000
C	0.514843000000	1.843106000000	-0.604797000000
S	-1.017253000000	-1.101514000000	0.936623000000

G–E(el) = 0.06239953 Hartree
Final single point energy E(CCSD(T)) = -591.348103846337 Hartree

Nudged Elastic Band (NEB) settings for structural determination of 7-tsA, 7-tsB, 10-tsA, and 10-tsB

Method type....climbing image
Threshold for climbing image....2.00e-02 Eh/Bohr
Free endpoints.... off
Tangent type....improved
Number of intermediate images....6
Number of images free to move....6
Spring type for image distribution....distance between adjacent images
Spring constant....energy weighted (0.0100 -to- 0.1000) Eh/Bohr^2
Spring force perp. to the path....none
Generation of initial path....image dependent pair potential
Initial path via TS guess....off

Minimization of RMSD: Rotation....always
Minimization of RMSD: Translation type....centroid
Center fixed to origin....true
Remove external force....true

Reparametrization of the path....off
Convergence thresholds:
Convergence monitored for....all images
Scaling factor....10.00

Convergence parameters for regular images:
    Max(|Fp|)....2.00e-02 Eh/Bohr
    RMS(Fp)....1.00e-02 Eh/Bohr

Convergence parameters for climbing image:
    Max(|F|)....2.00e-03 Eh/Bohr
    RMS(F)....1.00e-03 Eh/Bohr
Optimization method:
Method....L-BFGS
Max. iterations....500
Step size....1.00
Maximum allowed step size....0.10 Bohr

LBFGS parameters:

Memory....20
Initial step size....0.0010
Estimate curvature....YES
Reset on maxmove....YES
Reparam. on reset....NO

Summary of Natural Population Analysis at CCSD(T)/def2-TZVPP//PBE0/def2-TZVP

Alkylidene carbene 7

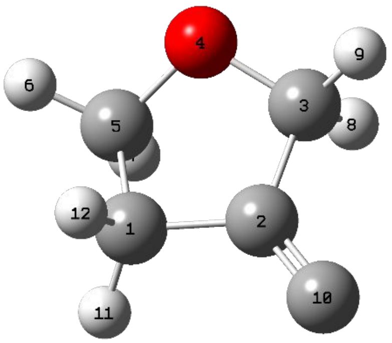

Summary of Natural Population Analysis:

Atom	No	Natural Charge	Natural Population Core	Valence	Rydberg	Total
C	1	-0.48484	1.99876	4.47870	0.00738	6.48484
C	2	-0.45463	1.99878	4.43559	0.02026	6.45463
C	3	-0.07356	1.99870	4.06379	0.01107	6.07356
O	4	-0.59122	1.99972	6.58723	0.00428	8.59122
C	5	-0.09025	1.99874	4.07992	0.01159	6.09025
H	6	0.23667	0.00000	0.76254	0.00079	0.76333
H	7	0.20403	0.00000	0.79367	0.00229	0.79597
H	8	0.21981	0.00000	0.77801	0.00218	0.78019
H	9	0.24648	0.00000	0.75256	0.00097	0.75352
C	10	0.26175	1.99941	3.72677	0.01207	5.73825
H	11	0.26129	0.00000	0.73763	0.00109	0.73871
H	12	0.26447	0.00000	0.73403	0.00150	0.73553
* Total *		-0.00000	11.99411	31.93042	0.07547	44.00000

7-tsA

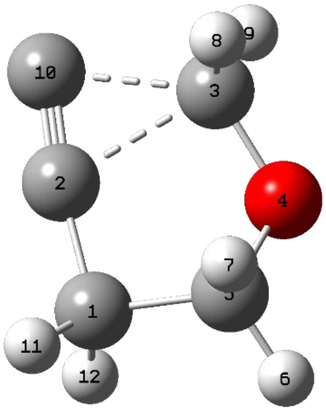

Summary of Natural Population Analysis:

Atom	No	Natural Charge	Core	Valence	Rydberg	Total
			Natural Population			
C	1	-0.53674	1.99865	4.52857	0.00952	6.53674
C	2	-0.15651	1.99857	4.14405	0.01390	6.15651
C	3	-0.03993	1.99893	4.02543	0.01556	6.03993
O	4	-0.59144	1.99967	6.58719	0.00458	8.59144
C	5	-0.06823	1.99876	4.05770	0.01177	6.06823
H	6	0.24022	0.00000	0.75901	0.00077	0.75978
H	7	0.20846	0.00000	0.78929	0.00225	0.79154
H	8	0.22376	0.00000	0.77363	0.00262	0.77624
H	9	0.27047	0.00000	0.72874	0.00080	0.72953
C	10	-0.09174	1.99899	4.07757	0.01518	6.09174
H	11	0.27163	0.00000	0.72719	0.00118	0.72837
H	12	0.27006	0.00000	0.72837	0.00157	0.72994
* Total *		-0.00000	11.99357	31.92673	0.07970	44.00000

7-tsB

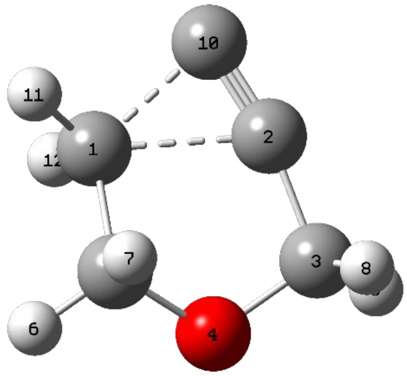

Summary of Natural Population Analysis:

Atom	No	Natural Charge	Natural Population Core	Valence	Rydberg	Total
C	1	-0.47484	1.99881	4.46723	0.00880	6.47484
C	2	-0.10416	1.99849	4.09292	0.01275	6.10416
C	3	-0.11704	1.99858	4.10520	0.01326	6.11704
O	4	-0.58874	1.99970	6.58460	0.00444	8.58874
C	5	-0.10583	1.99866	4.09466	0.01251	6.10583
H	6	0.24793	0.00000	0.75108	0.00099	0.75207
H	7	0.21110	0.00000	0.78645	0.00245	0.78890
H	8	0.22600	0.00000	0.77172	0.00228	0.77400
H	9	0.25403	0.00000	0.74475	0.00121	0.74597
C	10	-0.10362	1.99878	4.08887	0.01597	6.10362
H	11	0.28420	0.00000	0.71461	0.00118	0.71580
H	12	0.27098	0.00000	0.72706	0.00196	0.72902
* Total *		-0.00000	11.99302	31.92916	0.07782	44.00000

Alkylidene carbene 10

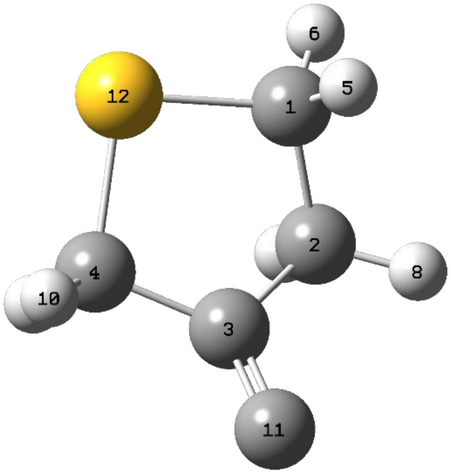

Summary of Natural Population Analysis:

Atom	No	Natural Charge	Natural Population Core	Valence	Rydberg	Total
C	1	-0.62243	1.99888	4.61625	0.00730	6.62243
C	2	-0.45701	1.99873	4.44830	0.00998	6.45701
C	3	-0.41772	1.99867	4.39799	0.02105	6.41772
C	4	-0.61299	1.99881	4.60552	0.00866	6.61299
H	5	0.26105	0.00000	0.73665	0.00230	0.73895
H	6	0.26898	0.00000	0.72971	0.00131	0.73102
H	7	0.26129	0.00000	0.73698	0.00173	0.73871
H	8	0.26589	0.00000	0.73280	0.00132	0.73411
H	9	0.27237	0.00000	0.72564	0.00200	0.72763
H	10	0.27597	0.00000	0.72224	0.00179	0.72403
C	11	0.28472	1.99941	3.70364	0.01224	5.71528
S	12	0.21989	9.99933	5.76355	0.01722	15.78011
* Total *		-0.00000	19.99383	31.91928	0.08689	52.00000

10-tsA

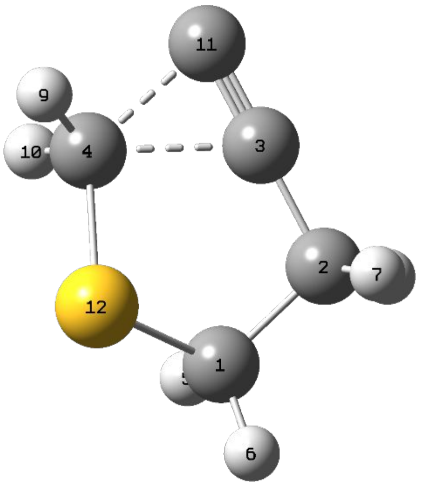

Summary of Natural Population Analysis:

Atom	No	Natural Charge	Natural Population Core	Valence	Rydberg	Total
C	1	-0.62136	1.99891	4.61489	0.00757	6.62136
C	2	-0.50943	1.99864	4.50022	0.01057	6.50943
C	3	-0.10296	1.99860	4.09063	0.01373	6.10296
C	4	-0.64752	1.99900	4.64062	0.00790	6.64752
H	5	0.26049	0.00000	0.73708	0.00242	0.73951
H	6	0.27360	0.00000	0.72534	0.00106	0.72640
H	7	0.27390	0.00000	0.72448	0.00162	0.72610
H	8	0.28070	0.00000	0.71814	0.00116	0.71930
H	9	0.30509	0.00000	0.69383	0.00107	0.69491
H	10	0.27824	0.00000	0.71936	0.00240	0.72176
C	11	-0.05015	1.99898	4.03513	0.01605	6.05015
S	12	0.25941	9.99927	5.72378	0.01754	15.74059
* Total *		0.00000	19.99338	31.92352	0.08310	52.00000

10-tsB

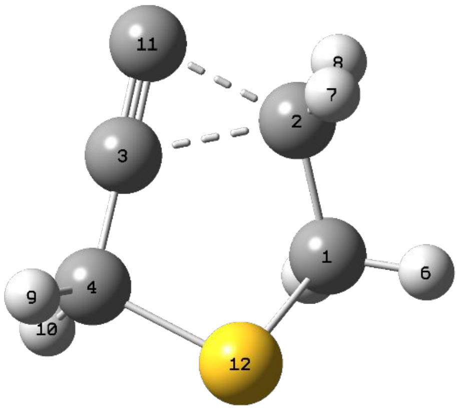

Summary of Natural Population Analysis:

Atom	No	Natural Charge	Natural Population Core	Valence	Rydberg	Total
C	1	-0.64951	1.99882	4.64286	0.00783	6.64951
C	2	-0.44654	1.99879	4.43725	0.01050	6.44654
C	3	-0.12963	1.99856	4.11669	0.01439	6.12963
C	4	-0.66331	1.99871	4.65404	0.01055	6.66331
H	5	0.26497	0.00000	0.73250	0.00253	0.73503
H	6	0.28213	0.00000	0.71665	0.00122	0.71787
H	7	0.27218	0.00000	0.72590	0.00192	0.72782
H	8	0.29433	0.00000	0.70460	0.00106	0.70567
H	9	0.29531	0.00000	0.70342	0.00127	0.70469
H	10	0.27774	0.00000	0.72002	0.00224	0.72226
C	11	-0.05749	1.99898	4.04338	0.01514	6.05749
S	12	0.25982	9.99928	5.72409	0.01681	15.74018
* Total *		0.00000	19.99313	31.92139	0.08548	52.00000

References.

1. Neese, F., The ORCA program system. *Wiley Interdiscip. Rev. Comput. Mol. Sci.* **2012,** *2* (1), 73-78.
2. Neese, F., Software update: the ORCA program system, version 4.0. *Wiley Interdiscip. Rev. Comput. Mol. Sci.* **2018,** *8*.
3. Neese, F., Software update: The ORCA program system—Version 5.0. *Wiley Interdiscip. Rev. Comput. Mol. Sci.* **2022,** *n/a* (n/a), e1606.
4. Adamo, C.; Barone, V., Toward reliable density functional methods without adjustable parameters: The PBE0 model. *J. Chem. Phys.* **1999,** *110*, 6158-6170.
5. Weigend, F.; Ahlrichs, R., Balanced basis sets of split valence, triple zeta valence and quadruple zeta valence quality for H to Rn: Design and assessment of accuracy. *Phys. Chem. Chem. Phys.* **2005,** *7* (18), 3297-3305.
6. Guo, Y.; Sivalingam, K.; Valeev, E. F.; Neese, F., SparseMaps–A systematic infrastructure for reduced-scaling electronic structure methods. III. Linear-scaling multireference domain-based pair natural orbital N-electron valence perturbation theory. *J. Chem. Phys.* **2016,** *144* (9), 094111.
7. Hansen, A.; Liakos, D. G.; Neese, F., Efficient and accurate local single reference correlation methods for high-spin open-shell molecules using pair natural orbitals. *J. Chem. Phys.* **2011,** *135* (21), 214102.
8. Neese, F.; Hansen, A.; Liakos, D. G., Efficient and accurate approximations to the local coupled cluster singles doubles method using a truncated pair natural orbital basis. *J. Chem. Phys.* **2009,** *131* (6), 064103.
9. Neese, F.; Wennmohs, F.; Hansen, A., Efficient and accurate local approximations to coupled-electron pair approaches: An attempt to revive the pair natural orbital method. *J. Chem. Phys.* **2009,** *130* (11), 114108.
10. Pavošević, F.; Peng, C.; Pinski, P.; Riplinger, C.; Neese, F.; Valeev, E. F., SparseMaps-A systematic infrastructure for reduced scaling electronic structure methods. V. Linear scaling explicitly correlated coupled-cluster method with pair natural orbitals. *J. Chem. Phys.* **2017,** *146* (17), 174108.
11. Pinski, P.; Riplinger, C.; Valeev, E. F.; Neese, F., Sparse maps—A systematic infrastructure for reduced-scaling electronic structure methods. I. An efficient and simple linear scaling local MP2 method that uses an intermediate basis of pair natural orbitals. *J. Chem. Phys.* **2015,** *143* (3), 034108.
12. Riplinger, C.; Neese, F., An efficient and near linear scaling pair natural orbital based local coupled cluster method. *J. Chem. Phys.* **2013,** *138* (3), 034106.
13. Riplinger, C.; Pinski, P.; Becker, U.; Valeev, E. F.; Neese, F., Sparse maps–A systematic infrastructure for reduced-scaling electronic structure methods. II. Linear scaling domain based pair natural orbital coupled cluster theory. *J. Chem. Phys.* **2016,** *144* (2), 024109.
14. Riplinger, C.; Sandhoefer, B.; Hansen, A.; Neese, F., Natural triple excitations in local coupled cluster calculations with pair natural orbitals. *J. Chem. Phys.* **2013,** *139* (13), 134101.
15. Wennmohs, F.; Neese, F., A comparative study of single reference correlation methods of the coupled-pair type. *Chem. Phys.* **2008,** *343* (2), 217-230.
16. Weigend, F., Accurate Coulomb-fitting basis sets for H to Rn. *Phys. Chem. Chem. Phys.* **2006,** *8* (9), 1057-1065.

17. Chmela, J.; Harding, M. E., Optimized auxiliary basis sets for density fitted post-Hartree–Fock calculations of lanthanide containing molecules. *Mol. Phys.* **2018,** *116* (12), 1523-1538.
18. Hellweg, A.; Hättig, C.; Höfener, S.; Klopper, W., Optimized accurate auxiliary basis sets for RI-MP2 and RI-CC2 calculations for the atoms Rb to Rn. *Theor. Chem. Acc.* **2007,** *117* (4), 587-597.
19. Grimme, S.; Antony, J.; Ehrlich, S.; Krieg, H., A consistent and accurate ab initio parametrization of density functional dispersion correction (DFT-D) for the 94 elements H-Pu. *J. Chem. Phys.* **2010,** *132* (15), 154104.
20. Grimme, S.; Ehrlich, S.; Goerigk, L., Effect of the damping function in dispersion corrected density functional theory. *J. Comput. Chem.* **2011,** *32* (7), 1456-1465.
21. Perdew, J. P.; Burke, K.; Ernzerhof, M., Generalized Gradient Approximation Made Simple [Phys. Rev. Lett. 77, 3865 (1996)]. *Phys. Rev. Lett.* **1997,** *78* (7), 1396-1396.
22. Neese, F., An improvement of the resolution of the identity approximation for the formation of the Coulomb matrix. *J. Comput. Chem.* **2003,** *24* (14), 1740-1747.
23. Izsák, R.; Hansen, A.; Neese, F., The resolution of identity and chain of spheres approximations for the LPNO-CCSD singles Fock term. *Mol. Phys.* **2012,** *110* (19-20), 2413-2417.
24. Izsák, R.; Neese, F., An overlap fitted chain of spheres exchange method. *J. Chem. Phys.* **2011,** *135* (14), 144105.
25. Neese, F.; Wennmohs, F.; Hansen, A.; Becker, U., Efficient, approximate and parallel Hartree-Fock and hybrid DFT calculations. A 'chain-of-spheres' algorithm for the Hartree-Fock exchange. *Chem. Phys.* **2009,** *356*, 98-109.
26. Lee, T. J.; Taylor, P. R., A diagnostic for determining the quality of single-reference electron correlation methods. *Int. J. Quantum Chem.* **1989,** *36* (S23), 199-207.
27. Henkelman, G.; Uberuaga, B. P.; Jónsson, H., A climbing image nudged elastic band method for finding saddle points and minimum energy paths. *J. Chem. Phys.* **2000,** *113*, 9901-9904.
28. Henkelman, G. A.; Jónsson, H., Improved tangent estimate in the nudged elastic band method for finding minimum energy paths and saddle points. *J. Chem. Phys.* **2000,** *113*, 9978-9985.
29. Ásgeirsson, V.; Birgisson, B. O.; Bjornsson, R.; Becker, U.; Neese, F.; Riplinger, C.; Jónsson, H., Nudged Elastic Band Method for Molecular Reactions Using Energy-Weighted Springs Combined with Eigenvector Following. *J. Chem. Theory Comput.* **2021,** *17* (8), 4929-4945.
30. Fritsch, P., IV. Ueber die Darstellung von Diphenylacetaldehyd und eine neue Synthese von Tolanderivaten. *Liebigs Ann. Chem.* **1894,** *279* (3), 319-323.
31. Foster, J. M.; Boys, S. F., Canonical Configurational Interaction Procedure. *Rev. Mod. Phys.* **1960,** *32* (2), 300-302.
32. Dennington, R.; Keith, T. A.; Millam, J. M. *GaussView*, Version 6; Semichem Inc.: Shawnee Mission, KS, 2016.
33. Zhurko, G. A. *Chemcraft - Graphical Program for Visualization of Quantum Chemistry Computations*, Ivanovo, Russia: https://www.chemcraftprog.com, 2005.
34. ORCA Tutorials. https://www.orcasoftware.de/tutorials_orca/prop/thermo.html (accessed September 2022).

35. Carroll, F. A., Arrhenius Theory and Transition-State Theory. In *Perspectives on Structure and Mechanism in Organic Chemistry*, Brooks/Cole: Pacific Grove, 1998; p 344.
36. Bach, R. D., Ring Strain Energy in the Cyclooctyl System. The Effect of Strain Energy on [3 + 2] Cycloaddition Reactions with Azides. *J. Am. Chem. Soc.* **2009,** *131* (14), 5233-5243.
37. Takeda, T.; Sasaki, R.; Fujiwara, T., Carbonyl Olefination by Means of a *gem*-Dichloride−$Cp_2Ti[P(OEt)_3]_2$ System. *J. Org. Chem.* **1998,** *63* (21), 7286-7288.
